# Molecular Epidemiology of A/H3N2 and A/H1N1 Influenza Virus during a Single Epidemic Season in the United States

**DOI:** 10.1371/journal.ppat.1000133

**Published:** 2008-08-22

**Authors:** Martha I. Nelson, Laurel Edelman, David J. Spiro, Alex R. Boyne, Jayati Bera, Rebecca Halpin, Elodie Ghedin, Mark A. Miller, Lone Simonsen, Cecile Viboud, Edward C. Holmes

**Affiliations:** 1 Center for Infectious Disease Dynamics, Department of Biology, The Pennsylvania State University, University Park, Pennsylvania, United States of America; 2 Surveillance Data Inc., Plymouth Meeting, Pennsylvania, United States of America; 3 The J. Craig Venter Institute, Rockville, Maryland, United States of America; 4 Division of Infectious Diseases, University of Pittsburgh, Pittsburgh, Pennsylvania, United States of America; 5 Fogarty International Center, National Institutes of Health, Bethesda, Maryland, United States of America; 6 Department of Global Health, School of Public Health and Health Services, The George Washington University, Washington, D.C., United States of America; University of Wisconsin-Madison, United States of America

## Abstract

To determine the spatial and temporal dynamics of influenza A virus during a single epidemic, we examined whole-genome sequences of 284 A/H1N1 and 69 A/H3N2 viruses collected across the continental United States during the 2006–2007 influenza season, representing the largest study of its kind undertaken to date. A phylogenetic analysis revealed that multiple clades of both A/H1N1 and A/H3N2 entered and co-circulated in the United States during this season, even in localities that are distant from major metropolitan areas, and with no clear pattern of spatial spread. In addition, co-circulating clades of the same subtype exchanged genome segments through reassortment, producing both a minor clade of A/H3N2 viruses that appears to have re-acquired sensitivity to the adamantane class of antiviral drugs, as well as a likely antigenically distinct A/H1N1 clade that became globally dominant following this season. Overall, the co-circulation of multiple viral clades during the 2006–2007 epidemic season revealed patterns of spatial spread that are far more complex than observed previously, and suggests a major role for both migration and reassortment in shaping the epidemiological dynamics of human influenza A virus.

## Author Summary

This study is the first of its kind to reconstruct the spread of an epidemic of influenza A virus across a single country, in this case the United States. In contrast to a single viral lineage spreading across this country, a phylogenetic analysis of the whole-genome sequences of more than 300 influenza A viruses of the A/H1N1 and A/H3N2 subtypes sampled from the 2006–2007 epidemic season reveals that multiple phenotypically and antigenically distinct viral lineages of entered and co-circulated in the US during this time. Furthermore, the widespread co-circulation of multiple lineages, even in geographically remote localities, allowed for frequent reassortment between influenza A viruses of the same subtype. Through reassortment, a minor lineage of A/H3N2 viruses surprisingly re-acquired sensitivity to the adamantane class of antiviral drugs, and a new A/H1N1 antigenic variant emerged that later became globally dominant. In sum, these results highlight the complexity of the spread of influenza A virus in time and space, and highlight the need for intensified global surveillance involving whole-genome sequence data.

## Introduction

Intensive study of the molecular evolution of influenza A virus has provided important insights into its seasonal genesis and spread in human populations [1]–[4]. The rapidity with which both epidemics and pandemics of influenza A virus arise and spread globally has also generated great interest in understanding the spatial-temporal dynamics of this important human pathogen [5]–[9]. Phylogenetic trees of the epitope-rich HA1 domain of subtype H3N2 influenza A viruses sampled since its emergence in 1968 exhibit a distinctive ‘cactus-like’ pattern, in which most lineages go extinct within a few years of their genesis, so that usually only a single lineage persists between seasonal epidemics [10],[11]. This is most likely the result of strong host-mediated selection pressure, resulting in continual evolution at key antigenic sites, a process termed ‘antigenic drift’ [11],[12]. This antigenic evolution is also episodic, with major changes in antigenicity occurring with a periodicity of approximately 3 years [13]. A variety of epidemiological and evolutionary models have been developed to explain this phylogenetic pattern [14],[15], and how the evolution of the HA1 domain relates to that in the rest of the viral genome [16].

Although antigenic drift is clearly a key determinant of influenza A virus evolution, this process has rarely been observed in a single locality over a single epidemic season [17],[18]. Rather, multiple viral introductions appear to drive evolution at the scale of local epidemics, allowing for the co-circulation of multiple clades of the same subtype [16],[18]. At a global scale, viral migration from regions characterized by more persistent influenza transmission, notably East and South-East Asia, appears to be important in determining large-scale epidemiological patterns [19],[20],[21]. In addition, reassortment events between viruses of the same subtype occur frequently, and are sometimes associated with major antigenic changes in both the A/H3N2 [22] and A/H1N1 subtypes [23]. However, a complete understanding of the evolutionary and epidemiologic dynamics of influenza A virus at all spatial and temporal scales remains an important goal [24].

Every winter, epidemics of human influenza recur in the United States, and are associated with an annual average of 226,000 hospitalizations and 36,000 deaths, mainly caused by secondary bacterial pneumonia in the elderly and young children [25],[26]. Epidemiological models have found a strong correlation between the regional spread of influenza virus infection in the United States and the movement of people to and from their workplace [9]. In addition, US influenza epidemics tend to originate in California, which may reflect this region's interconnectivity to Asia and Australia [9]. Although of great importance, most spatial models have utilized mortality cases due to pneumonia and influenza (P & I) and hence do not consider the evolutionary history of the viruses involved. Indeed, it is striking that detailed phylogenetic analyses of influenza A viruses from a single season at a national level have not been undertaken, even though the rapid rate of influenza A virus evolution [14], [27]–[29] means that viral genome sequences may contain important information on country-wide spatial dynamics.

Our goal here is to determine the spatial-temporal dynamics of influenza A virus during a single epidemic season (2006-2007) in the United States through the phylogenetic analysis of whole-genome sequence data. Since the 1968 pandemic, A/H3N2 viruses typically dominate most influenza seasons, including 16 of the past 20 US epidemics ([30], for example), and are associated with higher levels of morbidity and mortality [31], higher rates of evolutionary change [14], and greater synchrony in the timing of local epidemics across the United States than A/H1N1 viruses [9]. However, during the 2006–2007 US influenza epidemic, more viruses reported by the CDC were of the A/H1N1 (62.3%) than the A/H3N2 subtype (37.7%) [30]. The evolutionary dynamics of this epidemic were particularly complex, including a late-season switch in dominance from the A/H1N1 to the A/H3N2 subtype, the co-circulation of multiple antigenically distinct lineages within both A/H1N1 and A/H3N2, an A/H3N2 vaccine mismatch, and the co-circulation of adamantane resistant and sensitive viral lineages in both subtypes [30],[32]. Our analysis of 353 whole-genome influenza A virus sequences of both the A/H1N1 (n = 284) and A/H3N2 (n = 69) subtypes from this 2006–2007 US season represents the first attempt to investigate the spatial-temporal spread of a nationwide influenza virus epidemic within the context of genomic-scale evolutionary dynamics.

## Results

### Multiple introductions of A/H1N1 influenza virus during the 2006–2007 US season generate complex spatial patterns

**Figure 1:**
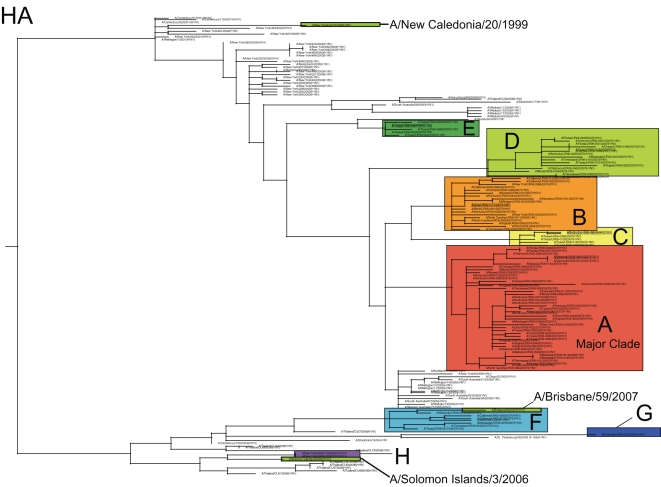
Phylogenetic relationships of the HA gene segment of 100 A/H1N1 influenza viruses sub-sampled from all eight clades that co-circulated in the United States during the 2006–2007 influenza season, 67 global isolates from 2001–2006, and the A/H1N1 component of the influenza vaccine used from the years 2000–2001 to 2006–2007 (A/New Caledonia/20/1999), the A/H1N1 strain selected for the 2007–2008 vaccine (A/Solomon Islands/3/2006), and the 2008–2009 A/H1N1 vaccine component, (A/Brisbane/59/2007), estimated using an ML method. Colored rectangles (labeled A–H) represent eight clades of related viral isolates from the 2006–2007 US season that are present on phylogenies for all eight viral genome segments (Figures S1, S2, S3, S4, S5, S6). Global background isolates are unshaded and labeled by season in red font. Vaccine strains are highlighted in olive green. Bootstrap values (>70%) are shown for key nodes. The tree is mid-point rooted for purposes of clarity only, and all horizontal branch lengths are drawn to scale.

**Figure 2 ppat-1000133-g002:**
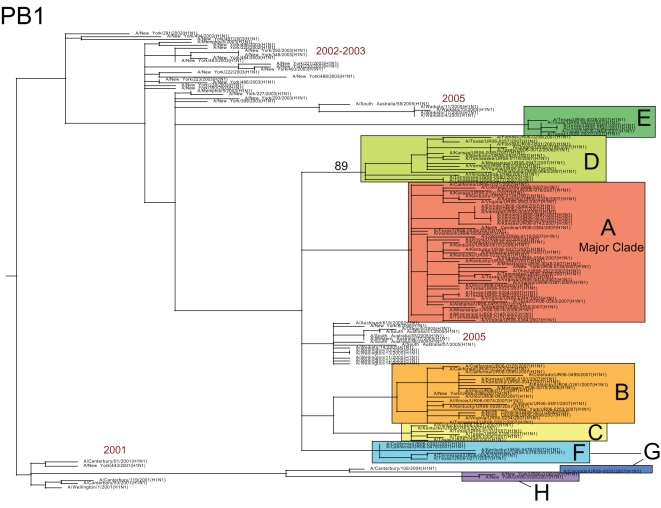
Phylogenetic relationships of the PB1 gene segment of 100 A/H1N1 influenza viruses sampled from the US during the 2006–2007 influenza season and 48 globally from 2001–2006, estimated using an ML method (representative of phylogenies for NP, M1/2, and NS1/2). Labels, shading, and rooting are the same as for Figure 1. Arrow indicates reassortant clade F.

Our phylogenetic analysis of 284 whole-genome A/H1N1 influenza viruses sampled between December 2006 and March 2007 in 17 US states revealed substantial genetic diversity for all eight segments of the viral genome. In particular, eight phylogenetically distinct clades (denoted A–H), defined by both high bootstrap values and long branch lengths, are evident on the trees of each genome segment, as exemplified by the HA phylogeny (Figure 1). The phylogenies of the seven other genome segments contain clades identical to those on the HA phylogeny (Figures S1, S2, S3, S4, S5, and S6, with the PB1 phylogeny presented in Figure 2). Previous studies [20], [22]–[23] suggest that each clade is likely to represent a separate introduction of the virus into the United States, although the small sample of sequences available mean that individual clades may sometimes represent multiple introduction events. One clade, herein denoted clade A, was clearly dominant, as it comprised the majority of isolates (175/284 isolates, 61.6%, Table 1). Minor clades B, C, D, E, F, G, and H contained only 47, 12, 35, 6, 6, 1, and 2 isolates each, respectively (Table 1).

**Table 1:** Number of A/H1N1 influenza viruses sampled per locality, per clade (Figure 1).

		Pop*	A	B	C	D	E	F	G	H	TOTAL
Major cities	New York City, NY	8214	2	2						2	6
	Los Angeles, CA	3849	5	7				2			14
	Chicago, IL	2833	12	3							15
	Houston, TX	2144	23	1	8	3	6	1			42
Mid-sized cities	Detroit, MI	871		1							1
	Denver, CO	567	8	1					1		10
	Nashville, TN	552				1					1
	Oklahoma City, OK	538	2			1					3
	Portland, OR	537				6					6
	Kansas City, MO	447	9	1		1					11
	Cleveland, OH	444	1	1							2
	Tampa, FL	333	8			4					12
	Cincinnati, OH	332	23	2	4			1			30
	Toledo, OH	298		1							1
	Birmingham, AL	229	1								1
	Akron, OH	210	1								1
	Winston-Salem, NC	197		1							1
	Richmond, VA	193	13	1							14
	Knoxville, TN	182	9			2					11
Minor cities/town	Albany, NY	94	12			2					14
	Madison, AL	36.8	1								1
	Pekin, IL	33.4	2	9							11
	Hopkinsville, KY	30.1	15	7		4					26
	Tullahoma, TN	18.9	1	2		8					11
	Dyersburg, TN	17.4	1					1			2
	Washington, OH	13.5	9	3							12
	Graham, NC	12.8	2	1							3
	Aberdeen, MS	6.4	9			1		1			14
	Weber City, VA	1.3	6	3		1					7
	Dunlap, IL	0.9				1					1
	TOTAL		175	47	12	35	6	6	1	2	284

Localities are listed in order of population size (^*^per 1,000 persons) and categorized as major cities (population of >1 million), mid-sized cities (population size of 100,000–999,999), or minor cities/towns (population of <99,999). Total number of isolates per locality listed in far right column.

**Figure 3 ppat-1000133-g003:**
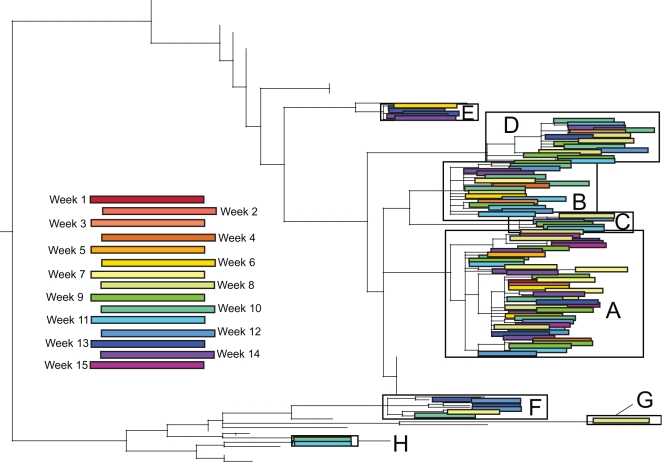
Temporal patterning of isolates contained in eight clades of A/H1N1 influenza viruses identified in Figure 1. Colored rectangles contain individual isolates that were collected during the week associated with that color (see Color Key). Dates for weeks 1–15 are the same as those used in Table 2. Branches leading to background isolates have been removed for clarity.

**Figure 4 ppat-1000133-g004:**
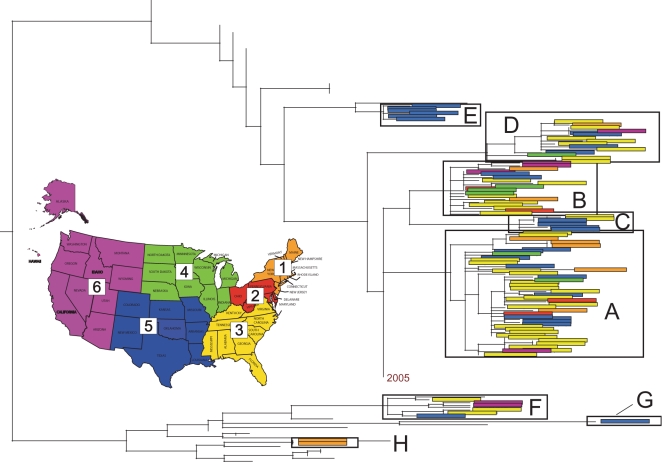
Spatial patterning of isolates contained in eight clades identified on the phylogeny of the HA gene segment of A/H1N1 influenza viruses from the 2006–2007 US epidemic that were identified in Figure 1. Colored rectangles contain individual isolates from the region of the United States associated with that color (see colored map): region 1 (Northeast) is orange, region 2 (Mid-Atlantic) is red, region 3 (South) is yellow, region 4 (Midwest) is green, region 5 (South Central) is blue, and region 6 (West) is purple. Branches leading to background isolates have been removed for clarity.

Clade A was the most geographically and temporally pervasive of the eight clades, circulating in 24/30 localities and 14/15 weeks studied, although allowall clades were sampled over wide temporal and geographic scales (Table 1, Figures 3, 4). Notably, there was no association between the phylogenetic positions of isolates and their week of collection (Figure 3) or geographic region (Figure 4). Rather, clades co-circulated in both time and space, with small clades that are detected in only a single region (E, G, and H) to likely be an artifact of limited sampling. The largest clades A and B were highly geographically dispersed, containing isolates collected from both relatively isolated areas and major US cities spanning all six US regions, including 24 and 18 out of 30 localities sampled, respectively (Table 1, Figure 4). However, in contrast to a simplified spatial model in which a single lineage spreads in a unidirectional manner, we observed no strong signal for viral migration among the co-circulating clades, even when individual clades were studied in isolation (Table 2). Indeed, a parsimony-based analysis in which the US state of origin of each isolate is coded as an extra character and mapped onto each ML tree revealed a strong clustering by US state (p<0.001), but only weak evidence for movement among states (data not shown; available from the authors on request).

**Table 2:** Clades of A/H1N1 (A–H, Figure 1) and A/H3N2 (a, b, s1, s2, s3, s4) influenza virus that co-circulate among multiple localities by epidemic week.

		Epidemic Week															
	°W	1	2	3	4	5	6	7	8	9	10	11	12	13	14	15	TOTAL
Portland, OR	122										Da	a	a	a			Da
Seattle, WA	122										a	a					a
Los Angeles, CA	118									Bs1	B	AB	AFa	ABFa	ABab		ABFabs1
Denver, CO	105						as2		G	A	Aa	Aa		Aa	ABa		ABGas2
Oklahoma City, OK	97								AD						A		AD
Houston, TX	95			D			AE	A		C	ACDF	ACD	ACa	AEab	ABEa	A	ABCDEFab
Kansas City, MO	94								AD	A	AB	A					ABD
Dunlap, IL	89									D							D
Dyersburg, TN	89							F	A								AF
Pekin, IL	89								AB	B	A						AB
Aberdeen, MS	88				A				AD	A		A	F		A	A	ADF
Chicago, IL	87					A		Aa		A	A	A	ABab	ABa	a		ABab
Hopkinsville, KY	87							D		A	ABDa	A	ADb	BD	B		ABDab
Birmingham, AL	86													a	A		Aa
Madison, AL	86														b		b
Nashville, TN	86														D		D
Tullahoma, TN	86								AD	BD		BD		D			ABD
Cincinnati, OH	84	A		A			AB	ABa	AC	AC	A	A	AC	F			ABCFa
Detroit, MI	83				B												B
Knoxville, TN	83									A		A	A	AD	AD		AD
Toledo, OH	83												B				B
Washington, OH	83									AB	AB		AB	Aa	A		ABa
Tampa, FL	82								A	a	D	D	A	A	Da	A	ADa
Weber City, VA	82								A	A	A	B					AB
Akron, OH	81											a		A			Aa
Cleveland, OH	81									B					A		AB
Winston-Salem, NC	80			B													B
Graham, NC	79									B			A				AB
Richmond, VA	77									AB	A	A	A		Aa	A	ABa
Washington, DC	77						a										a
Albany, NY	73							A	AD			D		Aa	Aa	A	ADa
New York City, NY	73							a	A	A	H	BH	Bb	a	as3s4		ABH
																	abs3s4
TOTAL		A		ABD	AB	A	ABE	ABDFa	ABCDG	ABCD	ABCDFHa	ABCDHa	ABCDFab	ABDEFab	ABDE	A	
							as2			as1					abs3s4		

Localities listed by longitudinal ordinates (°W) from west to east. The total number of clades that co-circulate in a given locality over the entire time period listed in the far right column. Week 1 begins 12/3/2006; week 2–12/10/06; week 3–12/17/06; week 4–12/24/06; week 5–12/31/06; week 6–1/7/2007; week 7–1/14/07; week 8–1/21/07; week 9–1/28/07; week 10–2/4/07; week 11–2/11/07; week 12–2/18/07; week 13–2/25/07; week 14–3/4/07; week 15–3/11/07.

The number of isolates collected from different US localities varied widely (ranging from 1 isolate from Detroit, Michigan to 42 isolates from Houston, Texas, Table 1), and such geographical biases in our data had a profound effect on spatial patterning. Accordingly, the number of clades identified in a locality was strongly associated with the number of isolates sampled from that locality (Spearman rho = 0.77, P<0.0001), while the population size of each locality was not associated with the number of viruses or clades identified (P>0.69). In addition, the first virus isolated in our A/H1N1 sample was from Cincinnati, Ohio (Table 2), likely an artifact of the relatively large sample collected from this city (30 isolates, Table 1).

The peak in A/H1N1 genetic diversity occurred during early February (corresponding to week 10, Table 2), with six of the eight clades co-circulating during this week. Geographically uneven sampling also meant that the most clades were detected in the most heavily sampled localities. For example, six clades (A, B, C, D, E, and F) were detected in Houston, Texas, the most intensively sampled locality (Table 1). Extensive genetic diversity was also detected within a single week: as a case in point, at least four clades (A, B, D, F), representing two major antigenically distinct lineages circulating globally (see below), were all present in Houston, Texas during week 10 (Table 2). Abundant viral diversity was also detected in localities that contributed relatively few (6–14) isolates, including both urban and remote areas. Three different clades circulated in all of the following localities: Los Angeles, California (clades A, C, F); Denver, Colorado (clades A, C, G); New York City, New York (clades A, C, H); Tullahoma, Tennessee (clades A, B, D), Aberdeen, Mississippi (clades A, D, F); and Weber City, Virginia (clades A, B, D) (Table 2). In fact, more than one clade was observed in every locality from which >1 viral sample was obtained (Table 2).

### At least three antigenically distinct clades of A/H1N1 virus co-circulated

To view the phylogenetic relationships among A/H1N1 clades from the 2006–2007 epidemic in a wider geographical context, we included 48 background A/H1N1 influenza viruses sampled from the northern and southern hemispheres between 2001–2006, years that were dominated by viruses antigenically similar to A/New Caledonia/20/1999 (‘New Caledonia-like’) [30]. These sequences were available for the HA and NA segments, including three antigenically distinct influenza vaccine reference strains selected for 2006–2007 (A/New Caledonia/20/1999), 2007–2008 (A/Solomon Islands/3/2006), and 2008–2009 (A/Brisbane/59/2007) (Figure 1) [30],[33],[34]. Of the eight clades that co-circulated during the 2006–2007 season, five (A, B, C, D, and E) appear to be descendents of New Caledonia-like viruses from 2002–2005, (Figure 1), while three (F, G, H) are separated from all other isolates by a very long branch with high (100%) bootstrap support (Figure 1). Due to their extensive phylogenetic divergence, we define clades F, G, and H as ‘set 2’ clades, in contrast to the ‘set 1’ clades A, B, C, D, and E.

**Figure 5:**
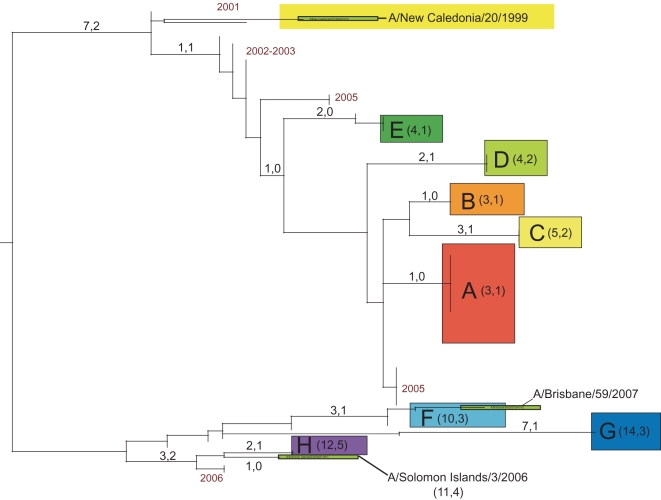
Number of amino acid changes along main branches of the tree depicting the phylogenetic relationships between the HA gene segments from A/H1N1 influenza viruses (adapted from Figure 1 with branches within clades collapsed). The first number represents total number of amino acid changes, with the number following comma referring to the number of amino acid changes occurring in antigenic or potential glycosylation sites. The number in parentheses next to the clade label represents the total number of amino acid changes and those changes at antigenic sites between that clade and the A/New Caledonia/20/1999 vaccine component used from 2000–2001 to 2006–2007.

We inferred the antigenic characteristics of these eight clades based on their phylogenetic relationships and the number of amino acid differences at antigenic sites in the HA from vaccine reference strains of known antigenicity. Accordingly, set 1 clades A, B, C, D, and E are likely to be New Caledonia-like in antigenicity, given that (a) 90% of A/H1N1 viruses from this US epidemic were New Caledonia-like (as characterized by the CDC surveillance [30]) and set 1 clades were most prevalent, (b) set 1 clades are phylogenetically related to other New Caledonia-like viruses from 2002–2005 (Figure 1), and (c) set 1 clades differ by only 3–5 amino acids from A/New Caledonia/20/1999, 1–2 of which occurred in antigenic or potential glycosylation sites, versus 10–14 amino acids, 3–5 in antigenic sites for set 2 clades (Figure 5, Table 3). It is possible that clades C and D represent additional antigenic variants of New Caledonia-like viruses, given the higher number of amino acid changes in antigenic sites (2) also observed in these viruses (Figure 5). However, given the uncertainties involved in inferring antigenic properties from genetic data alone, our antigenic assignments should not be considered definitive.

**Table 3:** Number of amino acid differences in the HA gene segment between clades A–H of A/H1N1 influenza virus sampled from the 2006–2007 US season.

	A	B	C	D	E	NC99	‘01	‘02/03	‘05	F	G	H	SI06
A	-	2	2^3^	3^4^	3	3^5^	2	2	3	**11^36^**	**15^36^**	**13^1223^**	**12^123^**
B	2	-	4^3^	3^4^	3	3^5^	2	2	3	**11^36^**	**15^36^**	**11^1223^**	**12^123^**
C	2^3^	4^3^	-	5^34^	5^3^	5^35^	4^3^	4^3^	5^3^	**9^6^**	**15^6^**	**13^122^**	**12^12^**
D	3^4^	3^4^	5^34^	-	4^4^	4^45^	3^4^	3^4^	4^4^	**12^346^**	**15^346^**	**14^12234^**	**13^1234^**
E	3	3	5^3^	4^4^	-	4^5^	3	3	4	**12^36^**	**16^36^**	**14^1223^**	**13^123^**
NC99	3^5^	3^5^	5^35^	4^45^	4^5^	-	1^5^	1^5^	4^5^	**10^356^**	**14^356^**	**12^12235^**	**11^1235^**
‘01	2	2	4^3^	3^4^	3	1^5^	-	0	3	**9^36^**	**13^36^**	**11^1223^**	**10^123^**
‘02/03	2	2	4^3^	3^4^	3	1^5^	0	-	3	**9^36^**	**13^36^**	**11^1223^**	**10^123^**
‘05	3	3	5^3^	4^4^	4	4^5^	3	3	-	**12^36^**	**16^36^**	**14^1223^**	**13^123^**
F	**11^36^**	**11^36^**	**9^6^**	**12^346^**	**12^36^**	**10^356^**	**9^36^**	**9^36^**	**12^36^**	-	9^6^	8^1226^	7^126^
G	**15^36^**	**15^36^**	**15^6^**	**15^346^**	**16^36^**	**14^356^**	**13^36^**	**13^36^**	**16^36^**	9^6^	-	10^1226^	7^126^
H	**13^1223^**	**11^1223^**	**13^1223^**	**14^12234^**	**14^1223^**	**12^12235^**	**11^1223^**	**11^1223^**	**14^1223^**	8^1226^	10^1226^	-	3^2^
SI06	**12^123^**	**12^123^**	**12^123^**	**13^1234^**	**13^123^**	**11^1235^**	**10^123^**	**10^123^**	**13^123^**	7^126^	7^126^	3^2^	-

As a comparison, isolates used as the A/H1N1 component of the influenza vaccine in 2006–2007 (A/New Caledonia/20/1999(H1N1) (NC99)) and 2007–2008 (A/Solomon Islands/3/2006(H1N1) (SI06)) are included, as well as representative isolates from the 2001 (‘01), 2002–2003 (‘02/03) and 2005 (‘05) seasons. Amino acid differences between clades in set 1 (clades A, B, C, D, E, NC99, ‘01, ‘02/03, and ‘05) and clades in set 2 (clades F, G, H, and SI06) are in bold. Amino acid changes in antigenic sites are denoted by superscripts as follows: ^1^–Cb antigenic site; ^2^–potential glycosylation site; ^3^–Ca_2_ antigenic site; ^4^–Sa antigenic site; ^5^–Ca_1_ antigenic site; ^6^–Sb antigenic site. For a complete list of amino acid changes at specific sites, see Table S1.

In contrast, set 2 clades F, G, and H appear to be related to two emerging antigenic variants. Clade H may be antigenically similar to the A/Solomon Islands/3/2006 vaccine strain selected for 2007–2008, based on their close phylogenetic relationship (Figure 1) and the low number of amino acid differences in antigenic sites (1 site, Table 3). Clade F is more phylogenetically related to the A/Brisbane/59/2007 2008–2009 vaccine strain, and there are no differences at antigenic sites in these viruses. Clades F and G differ by nine amino acids in the HA, but only one difference occurs at an antigenic site, suggesting that, although phylogenetically distinct, clade G may also be A/Brisbane/59/2007-like in antigenicity.

Also of note was the observation that of the 284 A/H1N1 influenza viruses sequenced in this study, only one isolate–A/Colorado/UR06-0053/2007–the sole member of clade G, contained the S31N amino acid replacement in the M2 protein that is associated with resistance to the adamantane class of antivirals (Table 4) [35].

**Table 4:** Summary of antigenic characterizations (based on phylogeny and amino acid comparisons with influenza A vaccine strains) and adamantane sensitivity (S31N) of all A/H1N1 and A/H3N2 clades detected in the United States during the 2006–2007 epidemic.

Clade	Subtype	Antigenic characterization	Adamantane sensitivity
A	A/H1N1	A/New Caledonia/20/1999-like	Sensitive
B	A/H1N1	A/New Caledonia/20/1999-like	Sensitive
C	A/H1N1	A/New Caledonia/20/1999-like	Sensitive
D	A/H1N1	A/New Caledonia/20/1999-like	Sensitive
E	A/H1N1	A/New Caledonia/20/1999-like	Sensitive
F	A/H1N1	A/Brisbane/59/2007-like	Sensitive
G	A/H1N1	A/Brisbane/59/2007-like	Resistant
H	A/H1N1	A/Solomon Islands/3/2006-like	Sensitive
a	A/H3N2	A/Brisbane/10/2007-like	Resistant
b	A/H3N2	Unknown	Sensitive
s1,s2,s3	A/H3N2	A/Wisconsin/67/2005-like	Resistant
s4	A/H3N2	Unknown	Sensitive

Antigenic characterizations inferred from phylogenetic relationships and differences in amino acids in antigenic sites of HA.

### Clade F was generated by intra-subtype reassortment between antigenic variants

Although clade F was classified as a member of clade set 2 due to the phylogenetic relatedness of its HA gene segment to clades G and H, this clade in fact appears to be set 1-set 2 reassortant. Specifically, on trees inferred for the PB2, PA, HA, and NA segments, clade F isolates are related to Solomon Islands-like set 2 clades G and H (as exemplified by the phylogeny of the HA gene segment, Figure 1). However, clade F is more closely related (with high bootstrap support) to the New Caledonia-like set 1 clades of A, B, C, and D in segments PB1, NP, M, and NS (as exemplified by phylogeny of PB1 gene segment, Figure 2; see Figures S1, S2, S3, S4, S5, and S6 for phylogenies of other segments). As half of the genome (PB1, NP, M, and NS) of these reassortant viruses was acquired from set 1-like viruses that began circulating in 2005, this reassortment event most likely occurred between 2005–2006.

### Multiple clades of both adamantane sensitive and resistant A/H3N2 influenza viruses co-circulated during the 2006–2007 US epidemic

**Figure 6:**
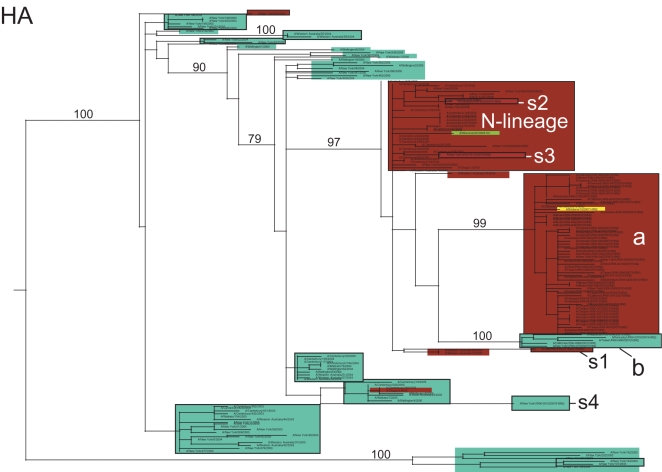
Phylogenetic relationships of the HA gene segment of 69 A/H3N2 influenza viruses sampled from the US during the 2006–2007 influenza season, 104 background global A/H3N2 viruses sampled from 2003–2006, and the A/H3N2 component of the influenza vaccines from the 2006–2007 and 2007–2008 seasons (A/Wisconsin/67/2005) and the component selected for the 2008–2009 influenza vaccine (A/Brisbane/10/2007). Isolates that are adamantane sensitive (have a serine at position 31 of the M2 gene) are shaded in blue, while isolates that are resistant to adamantane are shaded in red. Clades of related viral isolates are denoted by rectangles. Clades from the 2006–2007 US epidemic are labeled as follows: major clade (a), minor clade (b), and four singleton isolates (s1, s2, s3, and s4). Clades from previous global epidemics include the ‘N-lineage’ (adamantane-resistant isolates from 2005–2006). The A/Wisconsin/67/2005 vaccine strain, contained within the N-lineage, is shaded in olive, and the A/Brisbane/10/2007 vaccine strain, contained within clade a, is shaded in yellow. Bootstrap values (>70%) are shown for key nodes. The tree is mid-point rooted for purposes of clarity only, and all horizontal branch lengths are drawn to scale.

**Figure 7 ppat-1000133-g007:**
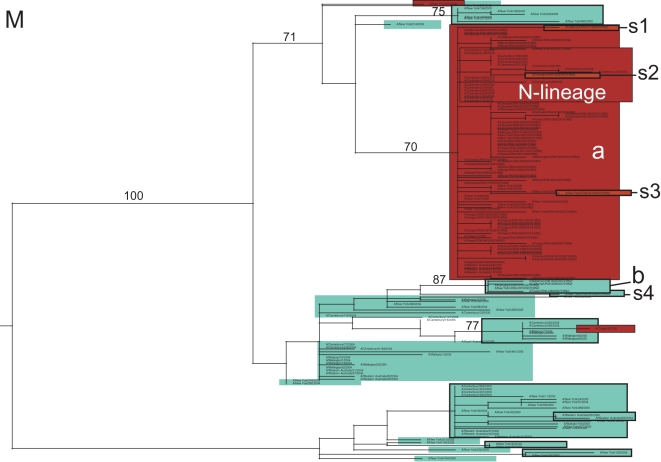
Phylogenetic relationships of the M gene segment of 69 A/H3N2 influenza viruses sampled from the US during the 2006–2007 influenza season and 104 background global A/H3N2 viruses sampled from 2003–2006. Clades, shading, labeling, and rooting are the same as in Figure 6.

Although fewer A/H3N2 influenza viruses (n = 69) were available for study due to the dominance of the A/H1N1 subtype during the 2006–2007 season, abundant genetic diversity is evident on all eight segment phylogenies, as exemplified by the HA tree (Figure 6; see Figures 7–9 and Figures S7, S8, S9, and S10 for trees of remaining segments). To obtain greater resolution, we also estimated phylogenetic trees that included 104 whole genome A/H3N2 influenza viruses sampled globally from 2003–2006 [36], as well as the HA and NA sequences from the A/H3N2 components of influenza vaccines selected for the 2006–2007/2007–2008 (A/Wisconsin/67/2005) and 2008–2009 (A/Brisbane/10/2007) seasons [30],[33],[34].

**Figure 8 ppat-1000133-g008:**
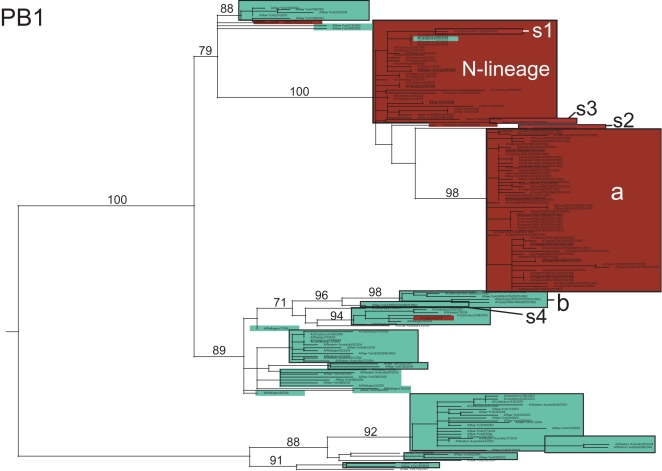
Phylogenetic relationships of the PB1 gene segment of 69 A/H3N2 influenza viruses sampled from the US during the 2006–2007 influenza season and 104 background global A/H3N2 influenza viruses sampled from 2003–2006. Clades, shading, labeling, and rooting are the same as in Figure 6.

**Figure 9:**
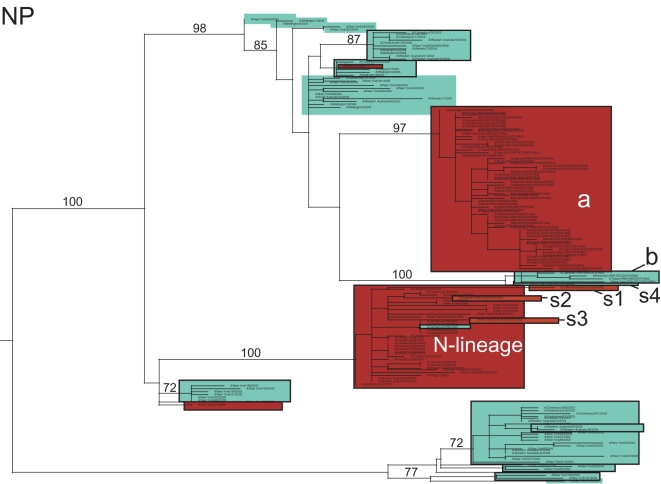
Phylogenetic relationships of the NP gene segment of 69 A/H3N2 influenza viruses sampled from the US during the 2006–2007 influenza season and 104 background global A/H3N2 influenza viruses sampled from 2003–2006. Clades, shading, labeling, and rooting are the same as in Figure 6.

The majority (60/69, 87.0%) of the A/H3N2 isolates from the 2006–2007 US epidemic were members of a major clade (denoted clade ‘a’). On both the HA and NA phylogenies, clade a contains the antigenically novel A/Brisbane/10/2007 isolate selected for the 2008–2009 vaccine, whereas only three 2006–2007 singleton isolates (i.e. isolates that were phylogenetically isolated; described below) belong to the clade that contains the 2006–2007 influenza vaccine strain A/Wisconsin/67/2005, confirming prior observations of a vaccine mismatch (Figure 6) [30]. This A/Wisconsin/67/2005-like clade first emerged in 2005 and represented a class of viruses that were adamantane-resistant due to the S31N mutation in M2; it was termed the ‘N-lineage’ in previous work [36]. This N-lineage is closely related to some 2003 isolates (previously termed ‘clade B’ [36]) in 4 of the 8 segment phylogenies (PB1, PA, NP, and M) (Figures 7, 8, 9, Figure S8), confirming that a 4+4 reassortment event was responsible for the genesis of the N-lineage [36]. As with the N-lineage, all isolates in clade a contained the S31N mutation in M2 that confers adamantane resistance (Figure 6).

In addition to the major clade a, a minor clade of five A/H3N2 viruses, denoted clade b, also circulated during the 2005–2006 season (Figure 6). Although clades a and b both descend from the adamantane resistant N-lineage, every isolate in clade b contains the adamantane-sensitive serine (S) at position 31 of the M2, indicating that a reversion has occurred. In addition, clades a and b may vary antigenically, as they differ in numerous amino acids in HA, five of which occur in antigenic sites A, B, and C (amino acid sites 50, 140, 142, 157, 173) and one–site 142–in the HA1 domain that was previously identified as undergoing positive selection [37].

Four singleton A/H3N2 viruses (labeled s1, s2, s3, and s4) also circulated during this season (Figure 6). Isolates s1, s2, and s3 are members of the older N-lineage and possess the associated adamantane-resistance S31N mutation. In contrast, isolate s4 is adamantane sensitive and clusters with other adamantane sensitive isolates, including clade b. The HA of isolate s4 differs from that of the major clade a by 12 amino acids, 8 of which occur at antigenic sites and 2 at previously identified positively selected sites (amino acid sites 193 and 275) [37] (Table 5). Similarly, the HA of s4 differs from clade b by 12 amino acids, 7 of which occur at antigenic sites and 2 at positively selected sites (142 and 193). In contrast, the HA of isolates s1, s2, and s3 differs from clade a by only 6, 2, and 4 amino acids in 3, 1, and 2 antigenic sites, respectively. In sum, as many as four antigenic variants of A/H3N2 influenza virus may have co-circulated this season (although this will be to be confirmed experimentally), each of which is likely to represent a separate introduction event: A/Wisconsin/67/2005-like (isolates s1, s2, and s3), A/Brisbane/10/2007-like (major clade a), clade b, and isolate s4 (Table 4).

**Table 5:** Amino acids at variable sites of the HA gene segment of A/H3N2 influenza viruses from the ‘N-lineage’, clade a, clade b, and singleton isolates s1, s2, s3, and s4 (Figure 6), with differing amino acids in bold.

HA site	N-lineage	s1	s2	s3	s4	Clade a	Clade b
6	N	N	N	N	N	N	**I**
45 (C)	S	S	S	S	**N**	S	S
48 (C)	T	T	T	T	**I**	T	T
50 (C)	G	G	**E**	G	G	**E**	G
53 (C)	D	D	D	D	**N**	D	D
112	V	V	V	V	**I**	V	V
140 (A)	K	K	K	K	K	**I**	K
142 (A*)	R	**G**	R	R	R	R	**G**
144 (A)	N	**D**	N	N	N	N	N
157 (B)	L	L	L	L	L	L	**S**
173 (D)	K	K	K	K	**E**	K	**E**
193 (B*)	F	F	F	F	**S**	F	F
199	S	S	S	S	**P**	S	S
225	N	N	N	N	**D**	N	N
275 (C*)	G	G	G	G	**D**	G	G
375	N	N	**D**	N	**D**	**D**	**D**
450	R	R	R	R	R	**K**	**K**

Antigenic sites are specified A–E (listed in parentheses) and sites that have been identified as undergoing positive selection denoted by an asterisk (^*^) [37].

### Multiple reassortment events involving A/H3N2 influenza viruses from the 2006–2007 US epidemic

Major topological differences between the eight phylogenies of the A/H3N2 virus genome strongly suggest that several reassortment events took place involving multiple clades from the 2006–2007 US epidemic. Whereas the adamantane-resistant clade a and the sensitive clade b both appear to derive from the N-lineage on the trees for the PB2, PA, HA, NA and NS segments, clade b instead derives from the adamantane sensitive clades from 2004–2005 on the M and PB1 trees (Figures 7, 8). This major phylogenetic incongruity strongly suggests that clade b viruses re-acquired sensitivity to adamantane by acquiring an older adamantane-sensitive M segment (with a serine at site 31 of the M2 gene) through reassortment. The NP segment also has undergone a major reassortment event, as on the NP phylogeny both clades a and b descend from adamantane sensitive clades, rather than from the N-lineage (Figure 9). The varying phylogenetic positions of the s4 isolate across the genome also suggest that this singleton virus resulted from multi-segment reassortment (Figures 6–9, Figures S7, S8, S9, S10). The s4 isolate is closely related to clade b on phylogenies of the PB2, PB1, NP, M, and NS segments (having reassorted along with clade b in the PB1 and M segments; Figures 7–9, Figures S7, S8, S9, S10). In contrast, on phylogenies of the PA, HA, and NA segments this virus is divergent from all other clades (Figure 6, Figures S7, S8, S9, S10).

### Spatial dynamics of A/H3N2 influenza viruses

No clear signal of the geographical spread of A/H3N2 influenza viruses could be detected due to our small sample size. All clades were geographically widespread and a secondary parsimony character mapping analysis again revealed strong population subdivision and weak migration (results not shown; available from authors upon request). The major clade a was present in all thirteen localities in which A/H3N2 viruses were collected, and the five isolates contained in minor clade b were geographically dispersed across both urban and remote areas spanning four of five US regions: Los Angeles, California; Chicago, Illinois; Hopkinsville, Kentucky; Madison, Alabama; New York City, New York; and Houston, Texas (Table 2). New York City exhibited the most A/H3N2 diversity, as major clade a, minor clade b, and singleton viruses s3 and s4 all were detected, which is remarkable given that only six total A/H3N2 isolates were collected from this locality (Table 2). In some cases, multiple clades of both A/H3N2 and A/H1N1 viruses co-circulated over restricted spatial-temporal scales. As a case in point, at least two A/H1N1 clades and two A/H3N2 clades circulated during week 12 in Chicago, Illinois, week 13 in Houston, Texas, and week 14 in Los Angeles, California (Table 2). Considering both A/H1N1 and A/H3N2 isolates together, large amounts of genetic diversity circulated in both urban and remote areas of the US: a total of 8 clades of influenza A virus circulated in Houston, Texas during the epidemic, 7 in New York City, New York, 6 in Los Angeles, California, and 5 clades each in Denver, Colorado, Cincinnati, Ohio, and Hopkinsville, Kentucky (Table 2).

## Discussion

This study utilized whole-genome sequence data from a surveillance initiative of unprecedented scope and scale that sampled both A/H1N1 and A/H3N2 influenza viruses across the US over the course of a single season through the Influenza Genomics Sequencing Project [38]. Rather than a single viral lineage spreading across the US, multiple lineages of both A/H3N2 and A/H1N1 influenza virus were separately introduced and co-circulated, allowing for reassortment within subtypes and greatly complicating patterns of spatial-temporal spread. Given the extent of genetic diversity observed during this season, obtaining a strong signal for the spatial-temporal pattern of spread of multiple different lineages clearly would entail a large increase in sampling.

Substantial antigenic diversity was also observed during the 2006–2007 season in the US, as at least five antigenically distinct types of influenza A virus co-circulated: three antigenically distinct variants of A/H1N1 viruses (A/New Caledonia/20/1999-like, A/Solomon Islands/3/2006-like, and A/Brisbane/59/2007-like), and at least two antigenically different types of A/H3N2 virus (A/Wisconsin/67/2005-like and A/Brisbane/10/2007-like), while clade b and isolate s4 also may represent additional antigenic variants of A/H3N2 virus (Table 4). However, analyses based on hemagglutinin-inhibition (HI) tests are required to confirm the antigenic status of these viruses.

Although A/Solomon Islands/3/2006-like viruses and A/Brisbane/59/2007-like A/H1N1 viruses were represented only by minor clades during the 2006–2007 season (H and F, respectively), Solomon Islands-like viruses achieved global A/H1N1 dominance by the start of the 2007–2008 season, and the reassortant clade of Brisbane-like viruses rose to dominance later during the 2007–2008 season [39]. Given that the antigenic evolution of A/H1N1 influenza virus is thought to be slower than the A/H3N2 virus, as reflected by eight consecutive years of dominance by A/New Caledonia/20/1999-like viruses, the rapid emergence of two new antigenic variants of A/H1N1 virus in a single year was particularly notable ([30], for example).

The extensive genetic diversity present in both A/H1N1 and A/H3N2 viruses suggests that multiple introductions of virus have taken place during the 2006–2007 season, particularly as our method of collecting viruses clearly underrepresented areas that are major ports of international travel. As a case in point, further sampling in the Los Angeles and New York City regions, where our study still detected significant diversity even at very low sampling levels, would likely augment the total number of viral lineages detected, including those imported from South-East Asia. By sampling in both metropolitan and relatively isolated areas, our study yielded important information on the geographic distribution of viral genetic variation: namely, that extensive viral diversity, including multiple antigenically distinguishable lineages, disseminated widely across the entire United States during the epidemic, even into relatively remote areas, so that it was not confined to the major cities where the virus is thought to enter. As a particular case in point, even relatively low-density areas or those distant from major metropolitan areas, such as Hopkinsville, Kentucky (population size ∼30,000), harbor significant amounts of both genetic and antigenic diversity, suggesting that influenza viruses of multiple antigenic (and other phenotypic) types extensively infiltrate the United States over the course of a single season. However, it is important to note that our analyses cannot exclude that a single co-infected individual could have introduced multiple clades of influenza virus into the United States, as the frequency of co-infection among patients in this study is unknown and represents a key area for further research.

Importantly, it is also possible that the 2006–2007 US epidemic was particularly difficult to reconstruct due to the unusual complexity of its evolutionary dynamics, which likely relates to the incomplete dominance of either the A/H1N1 or A/H3N2 subtype. The dynamics of influenza virus epidemics vary greatly on an annual basis, and influenza epidemics that are dominated by the A/H3N2 virus have been associated with higher disease transmission and more rapid spread than milder A/H1N1-dominated seasons, as well as stronger synchrony in timing across the United States [9]. Hence, epidemics that are dominated by a single A/H3N2 clade (such as the 2004–2005 season [18]) may exhibit stronger signals of spatial spread, and repeating this sampling effort during an A/H3N2-dominated influenza season potentially could yield a stronger spatial pattern. A sampling scheme that minimizes geographical biases and maximizes the number of samples collected early in the epidemic also could increase the likelihood of obtaining a stronger spatial signal.

Additional sequencing of influenza viruses in areas outside the United States is also essential to understand the global context of the diversity that enters the US during a given epidemic. From this, and previous studies [18],[21], it is clear that influenza A virus is introduced into the United States multiple times during an epidemic. However, the availability of global sequences, particularly at the genomic scale, is currently inadequate to draw any conclusions about the geographic origins of each viral introduction. It has been suggested that US epidemics originate more frequently in California than other states, due to high interconnectivity with Asia and Australia [9], but further whole-genome sequencing of viruses from Asia is clearly needed to test this hypothesis. Although the tendency of US epidemics to originate in the relatively warm state of California suggests that human movements are more important than climatic factors in the seasonal onset of influenza virus epidemics, further documentation of the complex spatial-temporal dissemination of the virus over an epidemic is required to elucidate the seasonality of influenza. Additionally, the extent of viral and antigenic diversity and the frequent circulation of minor clades that is detected by intensified surveillance efforts, such as the present study, suggest that much more diversity circulates at a global scale than is identified by routine surveillance. In particular, early detection of minor clades, particularly in the source populations of East and South-East Asia [21], could improve recognition of emerging lineages and prediction of future dominant strains for vaccine design. Indeed, the antigenically variant A/Brisbane/59/2007(H1N1)-like reassortant clade F detected in this study may not have been picked up by routine global surveillance until later, as no other publicly available global isolates from 2006 were found within this clade.

Our findings also suggest that the genetic diversity of the A/H3N2 virus is substantial even when A/H3N2 is not the dominant subtype, as was the case for most of the 2006–2007 epidemic. A major clade (a), a minor clade (b), a reassortant singleton (s4), and three singletons (s1, s2, s3) that appear to be descendents of the N-lineage [36] all co-circulated during this epidemic. All these clades differed in numerous amino acids in the HA, including those in antigenic and positively selected sites [37]. Both clades a and b, as well as the s4 singleton, were involved in at least three separate reassortment events: (a) clade b and singleton s4 (PB1 and M segments), (b) clades a and b (NP segment), and (c) singleton s4 only (PA, HA, and NA). As a caveat, because our study does not involve plaque-purified viruses, it is theoretically possible that the amplification of segments from different viruses co-infecting a single patient could produce a false signal for reassortment, particularly for those putative reassortment events that involve a single virus (for example, the s4 singleton). However, even with this potential source of bias, the frequency of definitive reassortment events among A/H3N2 clades is striking, especially compared to the single reassortment event observed among the A/H1N1 viruses that dominated this season. This most likely reflects the usually lower prevalence of A/H1N1, which in turn means a reduced likelihood of mixed infection and hence reassortment. In addition, given the importance of other geographical regions, particularly South-East Asia, in the evolution of the influenza A virus [21], as well as the fact that A/H3N2 was the dominant subtype in Canada and Europe during this season [33], the A/H3N2 virus likely circulated at higher levels outside the US, providing greater opportunity for reassortment. Of further interest is why inter-subtype reassortment between A/H1N1 and A/H3N2 viruses is not observed more commonly, despite the apparent co-circulation of both subtypes over both time and space (Table 2). In this case, it is possible that a virus produced by inter-subtype reassortment has a lower fitness, because the greater genetic distance between the A/H1N1 and A/H3N2 subtypes means that reassortment events are more likely to disrupt essential functional interactions among segments.

Finally, the existence of the adamantane-sensitive clade b (A/H3N2) during this epidemic was surprising, given that global resistance to adamantanes among influenza A/H3N2 viruses has increased dramatically in recent years, with more than 95% of A/H3N2 influenza viruses classified as resistant in the previous 2005–2006 season in the US [32]. Even more striking was that most of the genome of clade b isolates was more closely related to the adamantane-resistant clade a than to older adamantane-sensitive clades, indicating that this clade did not evolve directly from adamantane-sensitive viruses as may have been presumed. Rather, clade b viruses re-acquired sensitivity to adamantane by acquiring two segments (PB1 and M) from older adamantane-sensitive viruses through reassortment. This finding supports prior conclusions that sensitivity and resistance to adamantane can be acquired through genomic reassortment, rather than by direct selection on the M2 gene for drug resistant mutations [36].

## Methods

### Phylogenetic analysis of influenza A viruses from the 2006–2007 US epidemic

All viruses were collected as part of a larger 2006–2007 US surveillance effort conducted by Surveillance Data Inc., in which a total of 610 influenza virus specimens of both type A and type B were obtained from nasal and nasopharyngeal swabs from patients seen with influenza-like illness. At the time of this study, 353 type A influenza virus genomes had been sequenced and were available for study. Fifty-six participating physicians, primarily located at family practices, were recruited from 21 states that were geographically distributed across the US (AL, CA, CO, FL, IL, KS, KY, MI, MS, NJ, NY, NC, OH, OK, OR, PA, TN, TX, VT, VA, WA). Doctors swabbed all patients ≥ one year of age who presented with fever and upper respiratory symptoms from December 1, 2006 to April 1, 2007. An in-office immunoassay rapid test (Quidel QuickVue Influenza A+B Test) was used to identify positive influenza samples (A or B), and positive swabs were sent to a reference laboratory in Rochester, New York, to be typed as AH1N1, A/H3N2, or influenza B, following growth in Primary Rhesus Monkey Kidney Cells (RhMK) culture. RNA was extracted from viruses via automated nucleic acid extraction using the Roche MagNA Pure instrument and was shipped to the J. Craig Venter Institute in Rockville, Maryland, for whole virus sequencing (methods described previously [38]).

#### A/H1N1 influenza viruses

A total of 284 whole-genome sequences of A/H1N1 influenza virus sampled from the 2006–2007 season in the continental United States were used in this study (Table S2). A/H1N1 influenza viruses were collected from December 6, 2006 (which we regard as week 1 of the epidemic) to March 13, 2007, covering 15 weeks of the 2006–2007 epidemic. Isolates came from 33 localities within 18 US states, many of which are located within the greater metropolitan areas of larger cities (<50 miles away, listed in parentheses, when applicable): Columbiana, AL (Birmingham, AL), Madison, AL; Granada Hills, CA (Los Angeles, CA), Hacienda Heights, CA (Los Angeles, CA); Louisville, CO (Denver, CO); St. Petersburg, FL (Tampa, FL); Dunlap, IL, Naperville, IL (Chicago, IL), Pekin, IL; Overland Park, KS (Kansas City, MO); Florence, KY (Cincinnati, OH), Hopkinsville, KY; Royal Oak, MI (Detroit, MI); Aberdeen, MS; Bronx, NY (New York City, NY); Graham, NC (Durham, NC), Winston-Salem, NC; Fairfield, OH (Cincinnati, OH), North Canton, OH (Akron, OH), Oberlin, OH (Cleveland, OH), Toledo, OH, Washington, OH; Choctaw, OK (Oklahoma City, OK), Norman, OK (Oklahoma City, OK); Newberg, OR (Portland, OR); Dyersburg, TN, Knoxville, TN, Lebanon, TN (Nashville, TN) , Tullahoma, TN; Conroe, TX (Houston, TX); Richmond, VA, Weber City, VA; Bennington, VT (Albany, NY). Population sizes and longitudinal positions of these localities were determined using 2006 US Census data [40].

#### A/H3N2 influenza viruses

As A/H1N1 was dominant for most of the 2006–2007 US influenza season, fewer whole-genome sequences of A/H3N2 viruses were available for this study (69 isolates). However, as A/H3N2 replaced A/H1N1 as the dominant subtype towards the end of the 2006–2007 season in March, the majority (38/69, 55.1%) of A/H3N2 isolates used in this study were collected between February 25, 2007–March 10, 2007. These 69 A/H3N2 isolates came from 13 states representing all six US regions. Within these 13 states, isolates were sampled from 20 localities (those within the greater metropolitan areas of larger cities listed in parentheses, when applicable): Columbiana, AL (Birmingham, AL), Madison, AL; Hacienda Heights, CA (Los Angeles, CA); Louisville, CO (Denver, CO); St. Petersburg, FL (Tampa, FL); Bloomingdale, IL (Chicago, IL), Lake Zurich, IL (Chicago, IL), Naperville, IL (Chicago, IL); Florence, KY (Cincinnati, OH), Hopkinsville, KY; Bronx, NY (New York City, NY), Glendale, NY; Akron, OH, Washington, OH; Newberg, OR (Portland, OR); Conroe, TX (Houston, TX); Dale City, VA (Washington, DC), Richmond, VA; Bennington, VT (Albany, NY); Woodinville, WA (Seattle, WA).

#### Phylogenetic analysis

Genome sequences were downloaded from the NCBI Influenza Virus Resource [41]. Sequence alignments were manually constructed for the major coding regions of each of the eight genomic segments for both A/H1N1 and A/H3N2 viruses (with regions of overlapping reading frame deleted in the case of M1/2 and NS1/2): PB2 (2,277 nt), PB1 (2,271 nt), PA (2,148 nt), HA (1,698 nt), NP (1,494 nt), NA (1,407 nt), M1/2 (979 nt), NS1/2 (835 nt). Phylogenetic trees for the following A/H1N1 data sets were inferred using the maximum likelihood method available in PAUP* [42]: (a) each genome segment from all 284 A/H1N1 viruses available from the 2006–2007 US season; (b) a representative sub-set of 100 A/H1N1 viruses taken from all eight clades from the 2006–2007 season (Table S2), along with 48 global background sequences from 2001–2006 (largely from New York State, Australia, and New Zealand) for each genome segment (Table S3); (c) the sub-sample of 100 A/H1N1 and 48 global background HA sequences, plus 19 additional global HA sequences from 2006–2007, including the A/H1N1 isolates selected for the 2006–2007 (A/New Caledonia/20/1999), 2007–2008 (A/Solomon Islands/3/2006), and 2008–2009 (A/Brisbane/59/2007) vaccine strains [30],[33],[34] (total of 167 sequences) (Table S4); and (d) the sub-sample of 100 A/H1N1 NA and 48 global background NA sequences, plus 18 additional global NA sequences from 2006–2007, including the three influenza vaccine strains (A/New Caledonia/20/1999, A/Solomon Islands/3/2006, and A/Brisbane/59/2007) (total of 166 sequences) (Table S5). The only function of the background sequences was to put the phylogenetic relationships of the US sequences in a more global context.

For the A/H3N2 viruses, phylogenetic trees were inferred using the maximum likelihood method available in PAUP* [42] for each of the eight genome segments from 69 A/H3N2 viruses available sampled during the 2006–2007 US season (Table S6), and 104 whole genome A/H3N2 influenza viruses sampled globally from 2003–2006 that were studied previously [36] (total of 173 viruses) (Table S7). In addition, phylogenetic trees were estimated that included the HA and NA gene segments from the original 69 influenza viruses from 2006–2007 and the 104 viruses from 2003–2006, as well as HA and NA sequences from the A/H3N2 components of influenza vaccines produced for the 2006–2007/2007–2008 (A/Wisconsin/67/2005) and 2008–2009 (A/Brisbane/10/2007) seasons [30],[33],[34] (total of 175 sequences) (Table S8).

In each case, the best-fit model of nucleotide substitution was identified by MODELTEST [43] as the general reversible (GTR+I+Γ_4_) model, with the frequency of each substitution type, proportion of invariant sites (I), and the gamma distribution of among-site rate variation with four rate categories (Γ_4_) estimated from the empirical data (parameter values available upon request). In all cases tree bisection-reconnection (TBR) branch-swapping was utilized to determine the globally optimal tree. To assess the robustness of each node on the phylogenetic tree, a bootstrap resampling process (1,000 replications) using the neighbor-joining (NJ) method was used, incorporating the ML substitution model. Clades of related isolates were identified by high bootstrap values (>70) and exceptionally long branch lengths.

### Amino acid comparisons between clades of A/H1N1 and A/H3N2 influenza viruses

The parsimony-based MacClade program [44] was used to determine those amino acid changes in both the HA and NA gene segments (Table S9) that occurred between each of the eight clades of A/H1N1 virus from the US, as well as global background viruses from 2001–2005 and A/H1N1 vaccine strains. Changes were also identified in potential glycosylation sites, antigenic regions (Sa, Sb, Cb, Ca_1_, Ca_2_) [45], and the receptor-binding site [46]. The MacClade program also was employed to identify amino acid changes between clades of A/H3N2 and influenza virus vaccine strains, including those in antigenic sites and at eighteen sites previously identified as undergoing positive selection [37].

## Supporting Information

Figure S1 Phylogenetic relationships of the PB2 gene segment of A/H1N1 influenza viruses sampled from the United States during the 2006–2007 influenza season and globally from 2001–2006, estimated using an ML method. Colored rectangles (labeled A–H) represent eight clades of related viral isolates from the 2006–2007 U.S. season that are present on phylogenies for all genome segments (Figures S1, S2, S3, S4, S5, S6). Clade A represents the major clade from this season. Global background isolates are unshaded and labeled by season in red font. Bootstrap values (>70%) are shown for key nodes. The tree is mid-point rooted for clarity only, and all horizontal branch lengths are drawn to scale.
(0.50 MB EPS)

Figure S2 Phylogenetic relationships of the PA gene segment of A/H1N1 influenza viruses sampled from the United States during the 2006–2007 influenza season and globally from 2001–2006, estimated using an ML method. Color schemes and rooting are same as in Figure S1.
(0.45 MB EPS)

Figure S3 Phylogenetic relationships of the NP gene segment of A/H1N1 influenza viruses sampled from the United States during the 2006–2007 influenza season and globally from 2001–2006, estimated using an ML method. Color schemes and rooting are same as in Figure S1.
(0.49 MB EPS)

Figure S4 Phylogenetic relationships of the NA gene segment of A/H1N1 influenza viruses sampled from the United States during the 2006–2007 influenza season and globally from 2001–2006, estimated using an ML method. Color schemes and rooting are same as in Figure S1.
(0.49 MB EPS)

Figure S5 Phylogenetic relationships of the M gene segment of A/H1N1 influenza viruses sampled from the United States during the 2006–2007 influenza season and globally from 2001–2006, estimated using an ML method. Color schemes and rooting are same as in Figure S1.
(0.44 MB EPS)

Figure S6 Phylogenetic relationships of the NS gene segment of A/H1N1 influenza viruses sampled from the United States during the 2006–2007 influenza season and globally from 2001–2006, estimated using an ML method. Color schemes and rooting are same as in Figure S1.
(0.45 MB EPS)

Figure S7 Phylogenetic relationships of the PB2 gene segment of 69 A/H3N2 influenza viruses sampled from the United States during the 2006–2007 influenza season and 104 background global A/H3N2 influenza viruses sampled from 2003–2006. Isolates that are adamantane sensitive (have a serine at position 31 of the M2 gene) are shaded in blue, while isolates that are adamantane resistant are shaded in red. Clades of related viral isolates are denoted by rectangles. Clades from the 2006–2007 U.S. epidemic are labeled as follows: major clade (a), minor clade (b), and four singleton isolates (s1, s2, s3, and s4). Clades from previous global epidemics include the ‘N-lineage’ (adamantane-resistant isolates from 2005–2006). Bootstrap values (>70%) are shown for key nodes. The tree is mid-point rooted for purposes of clarity only, and all horizontal branch lengths are drawn to scale.
(0.43 MB EPS)

Figure S8 Phylogenetic relationships of the PA gene segment of 69 A/H3N2 influenza A viruses sampled from the United States during the 2006–2007 influenza season and 104 viruses sampled globally from 2003–2006. Labeling, color scheme, and rooting are identical as Figure S7.
(0.43 MB EPS)

Figure S9 Phylogenetic relationships of the NA gene segment of 69 A/H3N2 influenza A viruses sampled from the United States during the 2006–2007 influenza season and 104 viruses sampled globally from 2003–2006. Labeling, color scheme, and rooting are identical as Figure S7.
(0.47 MB EPS)

Figure S10 Phylogenetic relationships of the NS1/2 gene segment of 69 A/H3N2 influenza A viruses sampled from the United States during the 2006–2007 influenza season and 104 viruses sampled globally from 2003–2006. Labeling, color scheme, and rooting are identical as Figure S7.
(0.46 MB EPS)

Table S1 Amino acids at variable sites of the HA gene segment of A/H1N1 influenza viruses from clades A–H, the A/New Caledonia/20/1999(H1N1) and A/Solomon Islands/3/2006(H1N1) vaccine strains, and isolates from 2002/2003 and 2005 (Figure 1), with differing amino acids highlighted in bold. Antigenic sites Cb, Ca2, Sa, Ca1, Sb and potential glycosylation sites (Gly) listed in parentheses.
(0.10 MB DOC)

Table S2 Influenza A viruses used in Figures 1–5, and S1, S2, S3, S4, S5, S6. GenBank accession number, isolate name, subset membership, clade membership, date of collection, week of collection, age and sex of patient from whom isolate was collected, and county in which isolate was assembled for 284 A/H1N1 influenza A viruses collected from December 6, 2006–March 13, 2007 from 19 U.S. states. Subset 1 refers to those isolates included in the 100-isolate subset sampled from all clades; subset 2 refers to those isolates included in the 100-isolate subset sampled from the major clade. Week 1 denotes the first week that isolates in this study were sampled (week of December 2, 2006). GenBank accession numbers from the Influenza Virus Resource refer to the PB2 gene segment (http://www.ncbi.nlm.nih.gov/genomes/FLU/FLU.html). Clade membership corresponds to the HA phylogeny (Figure 1).
(0.49 MB DOC)

Table S3 Influenza A viruses used in Figures 1–5, and S1, S2, S3, S4, S5, S6. GenBank accession numbers, collection dates, and age and sex of patient from whom isolate were assembled for 48 A/H1N1 influenza viruses sampled globally from 2001–2006. GenBank accession numbers from the Influenza Virus Resource refer to the PB2 gene segment (http://www.ncbi.nlm.nih.gov/genomes/FLU/FLU.html).
(0.09 MB DOC)

Table S4 Influenza A viruses used in Figure 1. GenBank accession numbers and collection dates for the HA gene segment of 21 A/H1N1 influenza viruses sampled globally from 2006, including the A/H1N1 components of the influenza vaccines for 2006–2007 (A/New Caledonia/20.1999), 2007–2008 (A/Solomon Islands/3/2006), and 2008–2009 (A/Brisbane/59/2007). GenBank accession numbers from the Influenza Virus Resource refer to the PB2 gene segment (http://www.ncbi.nlm.nih.gov/genomes/FLU/FLU.html), and accession numbers starting with ISD are from the Los Alamos National Laboratory's Influenza Sequence Database (www.flu.lanl.gov).
(0.05 MB DOC)

Table S5 Influenza A viruses used in Figure S4. GenBank accession numbers and collection dates for the NA gene of 17 A/H1N1 influenza viruses sampled globally from 2006, including the A/H1N1 components of the influenza vaccines for 2006–2007 (A/New Caledonia/20/1999) and 2007–2008 (A/Solomon Islands/3/2006). GenBank accession numbers from the Influenza Virus Resource refer to the NA gene segment (http://www.ncbi.nlm.nih.gov/genomes/FLU/FLU.html).
(0.04 MB DOC)

Table S6 Influenza A viruses used in Figures 6–9 and Figures S7, S8, S9, S10. GenBank accession number, isolate name, subset membership, clade membership, date of collection, week of collection, age and sex of patient from whom isolate was collected, and county in which isolate were assembled for 69 A/H3N2 influenza A viruses collected from December 6, 2006–March 14, 2007 from 16 U.S. states. Week 1 denotes the first week that isolates in this study were sampled (week of December 2, 2006). GenBank accession numbers from the Influenza Virus Resource refer to the PB2 gene segment (http://www.ncbi.nlm.nih.gov/genomes/FLU/FLU.html).
(0.16 MB DOC)

Table S7 Influenza A viruses used in Figures 6–9 and Figures S7, S8, S9, S10. GenBank accession numbers, collection dates, and age and sex of patients from whom influenza viruses were assembled for 104 A/H3N2 influenza viruses sampled globally from 2003–2006. GenBank accession numbers from the Influenza Virus Resource refer to the PB2 gene segment (http://www.ncbi.nlm.nih.gov/genomes/FLU/FLU.html).
(0.17 MB DOC)

Table S8 A/H3N2 influenza viruses used in the 2006–2007/2007–2008 (A/Wisconsin/67/2005) and 2008–2009 (A/Brisbane/10/2007) influenza vaccines, the HA and NA gene segments of which are included in Figure 6. GenBank accession numbers from the Influenza Virus Resource refer to the PB2 gene segment (http://www.ncbi.nlm.nih.gov/genomes/FLU/FLU.html).
(0.03 MB DOC)

Table S9 Number of amino acid differences in the NA gene between clades A–H of A/H1N1 influenza virus from the 2006–2007 U.S. season. As comparison, isolates used as the H1N1 component of the influenza vaccine in 2006–2007 (A/New Caledonia/20/1999(H1N1) (NC99)) and 2007–2008 (A/Solomon Islands/3/2006(H1N1) (SI06)) are included. Amino acid differences between clades that cluster phylogenetically into set 1 (clades A–E and NC99) and clades that cluster phylogenetically into set 2 (clades F–H and SI06) are in bold.
(0.05 MB DOC)
