# Correction: Molecular Epidemiology of A/H3N2 and A/H1N1 Influenza Virus during a Single Epidemic Season in the United States

**DOI:** 10.1371/annotation/1391941e-93d3-48d3-8c9a-b7c6d98f9527

**Published:** 2008-12-15

**Authors:** Martha I. Nelson, Laurel Edelman, David J. Spiro, Alex R. Boyne, Jayati Bera, Rebecca Halpin, Naomi Sengamalay, Elodie Ghedin, Mark A. Miller, Lone Simonsen, Cecile Viboud, Edward C. Holmes

The authors wish to note the work of an additional author, Naomi Sengamalay, whose name was not published previously. Dr. Sengamalay is the 7th author on this paper, and was affiliated with affiliation number 3: The J. Craig Venter Institute, Rockville, Maryland, United States of America.

This author has a current address of: The Institute for Genome Sciences, University of Maryland School of Medicine, Baltimore, Maryland, United States of America.

The contributions of this author are as follows: Performed the experiments.

