# Supplementary material for: Molecular Epidemiology of A/H3N2 and A/H1N1 Influenza Virus during a Single Epidemic Season in the United States
Source: PLoS Pathog. 2008 Aug 22;4(8):e1000133. doi: 10.1371/journal.ppat.1000133 (PMC2495036; doi:10.1371/journal.ppat.1000133)
Supplement: Table S1 — Amino acids at variable sites of the HA gene segment of A/H1N1 influenza viruses from clades A–H, the A/New Caledonia/20/1999(H1N1) and A/Solomon Islands/3/2006(H1N1) vaccine strains, and isolates from 2002/2003 and 2005 (Figure 1), with differing amino acids highlighted in bold. Antigenic sites Cb, Ca2, Sa, Ca1, Sb and potential glycosylation sites (Gly) listed in parentheses. (0.10 MB DOC) [file ppat.1000133.s011.doc]

**Table S1**. Amino acids at variable sites of the HA gene segment of A/H1N1 influenza viruses from clades A-H, the A/New Caledonia/20/1999(H1N1) and A/Solomon Islands/3/2006(H1N1) vaccine strains, and isolates from 2002/2003 and 2005 (Figure 1), with differing amino acids highlighted in bold. Antigenic sites Cb, Ca_2_, Sa, Ca_1_, Sb and potential glycosylation sites (Gly) listed in parentheses.

| **Site** | **A** | **B** | **C** | **D** | **E** | **F** | **G** | **H** | **NC99** | **SI06** | **‘02/03** | **2005** |
| --- | --- | --- | --- | --- | --- | --- | --- | --- | --- | --- | --- | --- |
| 35 | Asp | Asp | Asp | Asp | Asp | **Asn** | Asp | Asp | Asp | Asp | Asp | Asp |
| 36 | Ser | Ser | Ser | **Arg** | Ser | Ser | **Asn** | Ser | Ser | Ser | Ser | Ser |
| 73 (Cb) | Lys | Lys | Lys | Lys | Lys | Lys | Lys | **Arg** | Lys | **Arg** | Lys | Lys |
| 82 | Thr | Thr | Thr | Thr | Thr | **Lys** | **Lys** | **Lys** | Thr | **Lys** | Thr | Thr |
| 94 | Tyr | Tyr | Tyr | Tyr | Tyr | **His** | **His** | **His** | Tyr | **His** | Tyr | Tyr |
| 121 | Ser | Ser | Ser | Ser | **Asn** | Ser | Ser | Ser | Ser | Ser | Ser | Ser |
| 128 (Gly) | Val | Val | Val | Val | Val | Val | Val | **Thr** | Val | **Thr** | Val | Val |
| 141 (Ca_2_) | Lys | Lys | **Glu** | Lys | Lys | **Glu** | **Glu** | **Glu** | Lys | **Glu** | Lys | Lys |
| 146 | Arg | Arg | Arg | Arg | Arg | Arg | **Lys** | **Lys** | Arg | **Lys** | Arg | Arg |
| 163 (Sa) | Lys | Lys | Lys | **Arg** | Lys | Lys | Lys | Lys | Lys | Lys | Lys | Lys |
| 166 (Ca_1_) | Ala | Ala | Ala | Ala | Ala | Ala | Ala | Ala | **Val** | Ala | Ala | Ala |
| 189 (Sb) | Arg | Arg | Arg | Arg | Arg | **Lys** | **Met** | Arg | Arg | Arg | Arg | Arg |
| 194 | Thr | Thr | Thr | Thr | Thr | Thr | **Lys** | Thr | Thr | **Lys** | Thr | Thr |
| 209 | Arg | Arg | Arg | Arg | Arg | **Lys** | **Lys** | **Lys** | Arg | **Lys** | Arg | Arg |
| 253 | Phe | Phe | Phe | Phe | Phe | **Tyr** | **Tyr** | **Tyr** | **Tyr** | **Tyr** | **Tyr** | Phe |
| 261 | Phe | Phe | Phe | Phe | **Leu** | Phe | Phe | Phe | Phe | Phe | Phe | Phe |
| 267 | Thr | Thr | Thr | Thr | Thr | **Asn** | **Asn** | **Asn** | Thr | **Asn** | Thr | Thr |
| 271 (Gly) | Pro | Pro | Pro | Pro | Pro | Pro | Pro | **Ser** | Pro | Pro | Pro | Pro |
| 273 | Asp | **Gly** | Asp | Asp | Asp | Asp | Asp | **Gly** | Asp | Asp | Asp | Asp |
| 271 | Pro | Pro | Pro | Pro | Pro | Pro | Pro | **Ser** | Pro | Pro | Pro | Pro |
| 273 | Asp | **Gly** | Asp | Asp | Asp | Asp | Asp | **Gly** | Asp | Asp | Asp | Asp |
| 274 | Glu | Glu | **Lys** | Glu | Glu | **Lys** | Glu | Glu | Glu | Glu | Glu | Glu |
| 310 | **Thr** | Ala | **Thr** | Ala | Ala | Ala | Ala | Ala | Ala | Ala | Ala | Ala |
| 315 | Val | Val | Val | Val | Val | Val | Val | Val | Val | Val | Val | **Ala** |
| 398 | Asn | Asn | Asn | Asn | Asn | Asn | Asn | Asn | Asn | Asn | Asn | **Ser** |
| 416 | Leu | Leu | Leu | Leu | Leu | **Ileu** | **Ileu** | **Ileu** | Leu | **Ileu** | Leu | Leu |
| 418 | Ileu | Ileu | Ileu | Ileu | Ileu | Ileu | **Val** | Ileu | Ileu | Ileu | Ileu | Ileu |
| 451 | Ser | Ser | Ser | Ser | Ser | Ser | **Asn** | Ser | Ser | Ser | Ser | Ser |
| 496 | Asn | Asn | Asn | Asn | Asn | Asn | **Ser** | Asn | Asn | Asn | Asn | Asn |
