# Supplementary material for: Molecular Epidemiology of A/H3N2 and A/H1N1 Influenza Virus during a Single Epidemic Season in the United States
Source: PLoS Pathog. 2008 Aug 22;4(8):e1000133. doi: 10.1371/journal.ppat.1000133 (PMC2495036; doi:10.1371/journal.ppat.1000133)
Supplement: Table S2 — Influenza A viruses used in Figures 1– 5, and S1, S2, S3, S4, S5, S6. GenBank accession number, isolate name, subset membership, clade membership, date of collection, week of collection, age and sex of patient from whom isolate was collected, and county in which isolate was assembled for 284 A/H1N1 influenza A viruses collected from December 6, 2006–March 13, 2007 from 19 U.S. states. Subset 1 refers to those isolates included in the 100-isolate subset sampled from all clades; subset 2 refers to those isolates included in the 100-isolate subset sampled from the major clade. Week 1 denotes the first week that isolates in this study were sampled (week of December 2, 2006). GenBank accession numbers from the Influenza Virus Resource refer to the PB2 gene segment (http://www.ncbi.nlm.nih.gov/genomes/FLU/FLU.html). Clade membership corresponds to the HA phylogeny (Figure 1). (0.49 MB DOC) [file ppat.1000133.s012.doc]

**Table S2**. Influenza A viruses used in Figures 1-5, S1-S6. GenBank accession number, isolate name, subset membership, clade membership, date of collection, week of collection, age and sex of patient from whom isolate was collected, and county in which isolate was assembled for 284 A/H1N1 influenza A viruses collected from December 6, 2006 – March 13, 2007 from 19 U.S. states. Subset 1 refers to those isolates included in the 100-isolate subset sampled from all clades; subset 2 refers to those isolates included in the 100-isolate subset sampled from the major clade. Week 1 denotes the first week that isolates in this study were sampled (week of December 2, 2006). GenBank accession numbers from the Influenza Virus Resource refer to the PB2 gene segment (<http://www.ncbi.nlm.nih.gov/genomes/FLU/FLU.html>). Clade membership corresponds to the HA phylogeny (Figure 1).

| **Accession** | **Isolate Name** | **Subsets** | **Clade** | **Collection Date (MM/DD/YY)** | **Week** | **Age of Patient (yrs)** | **Sex of Patient** | **County** |
| --- | --- | --- | --- | --- | --- | --- | --- | --- |
| ABV29776 | A/Alabama/UR06-0455/2007(H1N1) | 1,2 | A | 2/28/07 | 13 | 28 | M | Madison |
| ABW86452 | A/Alabama/UR06-0536/2007(H1N1) | 1,2 | A | 3/6/07 | 14 | 64 | F | Columbiana |
| ABW91624 | A/California/UR06-0125/2007(H1N1) | 1 | C | 1/30/07 | 9 | 5 | M | Hacienda Heights |
| ABW36244 | A/California/UR06-0232/2007(H1N1) | 1 | C | 2/9/07 | 10 | 4 | M | Hacienda Heights |
| ABW39849 | A/California/UR06-0302/2007(H1N1) |  | C | 2/14/07 | 11 | 5 | F | Hacienda Heights |
| ABW40168 | A/California/UR06-0321/2007(H1N1) | 1,2 | A | 2/16/07 | 11 | 44 | M | Hacienda Heights |
| ABW36189 | A/California/UR06-0374/2007(H1N1) | 2 | A | 2/20/07 | 12 | 46 | M | Hacienda Heights |
| ABW40047 | A/California/UR06-0375/2007(H1N1) | 2 | A | 2/20/07 | 12 | 6 | M | Hacienda Heights |
| ABW39860 | A/California/UR06-0393/2007(H1N1) | 1 | F | 2/21/07 | 12 | 5 | M | Granada Hills |
| ABV29765 | A/California/UR06-0435/2007(H1N1) |  | C | 2/26/07 | 13 | 6 | F | Hacienda Heights |
| ABX58271 | A/California/UR06-0440/2007(H1N1) | 2 | A | 2/27/07 | 13 | 7 | F | Hacienda Heights |
| ABY51258 | A/California/UR06-0442/2007(H1N1) |  | C | 2/26/07 | 13 | 3 | F | Granada Hills |
| ABY51049 | A/California/UR06-0479/2007(H1N1) | 1 | F | 3/1/07 | 13 | 13 | M | Hacienda Heights |
| ABY51269 | A/California/UR06-0552/2007(H1N1) | 2 | A | 3/8/07 | 14 | 6 | F | Granada Hills |
| ABW86386 | A/California/UR06-0564/2007(H1N1) |  | C | 3/7/07 | 14 | 30 | M | Hacienda Heights |
| ABW71381 | A/California/UR06-0585/2007(H1N1) | 1 | C | 3/9/07 | 14 | 11 | M | Hacienda Heights |
| ABW39838 | A/Colorado/UR06-0053/2007(H1N1) | 1 | G | 1/22/07 | 8 | 10 | M | Louisville |
| ABW39915 | A/Colorado/UR06-0110/2007(H1N1) | 1 | A | 1/30/07 | 9 | 9 | M | Louisville |
| ABV30381 | A/Colorado/UR06-0111/2007(H1N1) | 2 | A | 1/30/07 | 9 | 3 | F | Louisville |
| ABV29809 | A/Colorado/UR06-0207/2007(H1N1) | 2 | A | 2/7/07 | 10 | 6 | F | Louisville |
| ABV29710 | A/Colorado/UR06-0255/2007(H1N1) | 2 | A | 2/12/07 | 11 | 11 | F | Louisville |
| ABW40190 | A/Colorado/UR06-0287/2007(H1N1) | 2 | A | 2/13/07 | 11 | 12 | M | Louisville |
| ABW40245 | A/Colorado/UR06-0336/2007(H1N1) |  | A | 2/16/07 | 11 | 8 | F | Louisville |
| ABX58282 | A/Colorado/UR06-0496/2007(H1N1) |  | A | 3/1/07 | 13 | 2.5 | F | Louisville |
| ABV45969 | A/Colorado/UR06-0498/2007(H1N1) | 1 | A | 3/5/07 | 14 | 3 | M | Louisville |
| ABW71403 | A/Colorado/UR06-0499/2007(H1N1) | 1 | C | 3/5/07 | 14 | 5 | M | Louisville |
| ABW40630 | A/Florida/UR06-0049/2007(H1N1) | 1 | A | 1/22/07 | 8 | 12 | M | St Petersburg |
| ABW86331 | A/Florida/UR06-0208/2007(H1N1) | 1 | B | 2/7/07 | 10 | 6 | F | St Petersburg |
| ABY51093 | A/Florida/UR06-0209/2007(H1N1) |  | B | 2/7/07 | 10 | 3 | M | St Petersburg |
| ABV30304 | A/Florida/UR06-0280/2007(H1N1) |  | B | 2/14/07 | 11 | 15 | F | St Petersburg |
| ABW36200 | A/Florida/UR06-0354/2007(H1N1) | 2 | A | 2/20/07 | 12 | 6 | F | St Petersburg |
| ABV29985 | A/Florida/UR06-0355/2007(H1N1) | 2 | A | 2/20/07 | 12 | 5 | M | St Petersburg |
| ABV30315 | A/Florida/UR06-0383/2007(H1N1) | 2 | A | 2/21/07 | 12 | 14 | M | St Petersburg |
| ABW40113 | A/Florida/UR06-0412/2007(H1N1) | 2 | A | 2/26/07 | 13 | 3 | M | St Petersburg |
| ABW91305 | A/Florida/UR06-0447/2007(H1N1) |  | A | 2/27/07 | 13 | 12 | M | St Petersburg |
| ABW40025 | A/Florida/UR06-0501/2007(H1N1) | 1 | B | 3/5/07 | 14 | 13 | M | St Petersburg |
| ABW39871 | A/Florida/UR06-0577/2007(H1N1) | 1 | A | 3/13/07 | 15 | 5 | M | St Petersburg |
| ABW91327 | A/Florida/UR06-0578/2007(H1N1) |  | A | 3/13/07 | 15 | 10 | F | St Petersburg |
| ABV29589 | A/Illinois/UR006-018/2007(H1N1) | 1,2 | A | 1/4/07 | 5 | 8 | F | Naperville |
| ABV29578 | A/Illinois/UR06-0019/2007(H1N1) | 2 | A | 1/5/07 | 5 | 4 | M | Naperville |
| ABW91569 | A/Illinois/UR06-0032/2007(H1N1) | 1,2 | A | 1/16/07 | 7 | 32 | M | Naperville |
| ABW91591 | A/Illinois/UR06-0074/2007(H1N1) | 1 | C | 1/25/07 | 8 | 9 | M | Perin |
| ABW91382 | A/Illinois/UR06-0088/2007(H1N1) | 2 | A | 1/26/07 | 8 | 7 | M | Perin |
| ABX58513 | A/Illinois/UR06-0093/2007(H1N1) |  | C | 1/29/07 | 9 | 11 | F | Perin |
| ABW86485 | A/Illinois/UR06-0094/2007(H1N1) |  | C | 1/29/07 | 9 | 7 | F | Perin |
| ABW40454 | A/Illinois/UR06-0095/2007(H1N1) |  | C | 1/29/07 | 9 | 8 | M | Perin |
| ABW86397 | A/Illinois/UR06-0098/2007(H1N1) |  | A | 1/29/07 | 9 | 7 | F | Naperville |
| ABW86551 | A/Illinois/UR06-0115/2007(H1N1) |  | C | 1/30/07 | 9 | 6 | M | Naperville |
| ABW71469 | A/Illinois/UR06-0116/2007(H1N1) |  | C | 1/30/07 | 9 | 7 | M | Perin |
| ABW91426 | A/Illinois/UR06-0131/2007(H1N1) |  | C | 1/31/07 | 9 | 9 | M | Perin |
| ABW86430 | A/Illinois/UR06-0136/2007(H1N1) |  | C | 2/1/07 | 9 | 12 | M | Perin |
| ABW40619 | A/Illinois/UR06-0137/2007(H1N1) |  | C | 2/1/07 | 9 | 12 | M | Perin |
| ABW40553 | A/Illinois/UR06-0146/2007(H1N1) | 1 | B | 2/1/07 | 9 | 12 | M | Dunlap |
| ABV45892 | A/Illinois/UR06-0223/2007(H1N1) | 2 | A | 2/8/07 | 10 | 6 | M | Naperville |
| ABW40069 | A/Illinois/UR06-0224/2007(H1N1) |  | A | 1/6/07 | 5 | 37 | M | Naperville |
| ABW91547 | A/Illinois/UR06-0227/2007(H1N1) |  | A | 2/9/07 | 10 | 10 | F | Perin |
| ABV30029 | A/Illinois/UR06-0248/2007(H1N1) |  | A | 2/12/07 | 11 | 14 | M | Naperville |
| ABW86375 | A/Illinois/UR06-0249/2007(H1N1) | 2 | A | 2/12/07 | 11 | 25 | F | Naperville |
| ABV29853 | A/Illinois/UR06-0333/2007(H1N1) |  | C | 2/19/07 | 12 | 40 | M | Naperville |
| ABW39959 | A/Illinois/UR06-0376/2007(H1N1) | 2 | A | 2/21/07 | 12 | 41 | M | Naperville |
| ABV29699 | A/Illinois/UR06-0377/2007(H1N1) | 2 | A | 2/21/07 | 12 | 37 | F | Naperville |
| ABV30062 | A/Illinois/UR06-0415/2007(H1N1) |  | C | 2/26/07 | 13 | 30 | M | Naperville |
| ABV29963 | A/Illinois/UR06-0456/2007(H1N1) | 2 | A | 2/28/07 | 13 | 44 | F | Naperville |
| ABX58293 | A/Illinois/UR06-0475/2007(H1N1) |  | A | 3/1/07 | 13 | 13 | M | Naperville |
| ABW40212 | A/Illinois/UR06-0491/2007(H1N1) | 1 | C | 3/1/07 | 13 | 33 | M | Naperville |
| ABW91481 | A/Kansas/UR06-0068/2007(H1N1) |  | A | 1/24/07 | 8 | 4 | F | Overland Park |
| ABX58436 | A/Kansas/UR06-0084/2007(H1N1) |  | A | 1/26/07 | 8 | 8 | F | Overland Park |
| ABW91613 | A/Kansas/UR06-0085/2007(H1N1) | 1 | B | 1/26/07 | 8 | 7 | F | Overland Park |
| ABW40542 | A/Kansas/UR06-0104/2007(H1N1) |  | A | 1/30/07 | 9 | 8 | M | Overland Park |
| ABV30568 | A/Kansas/UR06-0140/2007(H1N1) | 2 | A | 2/1/07 | 9 | 3 | M | Overland Park |
| ABW91635 | A/Kansas/UR06-0143/2007(H1N1) | 1,2 | A | 1/30/07 | 9 | 12 | F | Overland Park |
| ABX58612 | A/Kansas/UR06-0191/2007(H1N1) | 1 | C | 2/5/07 | 10 | 6 | M | Overland Park |
| ABY51126 | A/Kansas/UR06-0192/2007(H1N1) | 1,2 | A | 2/5/07 | 10 | 5 | F | Overland Park |
| ABW86474 | A/Kansas/UR06-0283/2007(H1N1) | 2 | A | 2/12/07 | 11 | 5 | M | Overland Park |
| ABX58392 | A/Kansas/UR06-0284/2007(H1N1) |  | A | 2/12/07 | 11 | 4 | F | Overland Park |
| ABY81414 | A/Kansas/UR06-0299/2007(H1N1) | 2 | A | 2/14/07 | 11 | 5 | M | Overland Park |
| ABX58689 | A/Kentucky/UR06-0007/2006(H1N1) | 1 | A | 12/6/06 | 1 | 3 | M | Florence |
| ABV29567 | A/Kentucky/UR06-0010/2006(H1N1) | 1 | A | 12/18/06 | 3 | 8 | M | Florence |
| ABW40014 | A/Kentucky/UR06-0027/2007(H1N1) | 1,2 | A | 1/12/07 | 6 | 4 | M | Florence |
| ABV45947 | A/Kentucky/UR06-0028/2007(H1N1) | 1 | C | 1/12/07 | 6 | 8 | M | Florence |
| ABW71392 | A/Kentucky/UR06-0029/2007(H1N1) | 2 | A | 1/12/07 | 6 | 10 | M | Florence |
| ABV30326 | A/Kentucky/UR06-0033/2007(H1N1) | 2 | A | 1/15/07 | 7 | 2 | M | Florence |
| ABV30513 | A/Kentucky/UR06-0034/2007(H1N1) | 2 | A | 1/16/07 | 7 | 8 | F | Florence |
| ABV29930 | A/Kentucky/UR06-0042/2007(H1N1) | 1 | C | 1/18/07 | 7 | 11 | F | Florence |
| ABV30205 | A/Kentucky/UR06-0043/2007(H1N1) | 1 | A | 1/18/07 | 7 | 3 | F | Florence |
| ABX58469 | A/Kentucky/UR06-0046/2007(H1N1) |  | B | 1/19/07 | 7 | 6 | M | Hopkinsville |
| ABW39827 | A/Kentucky/UR06-0057/2007(H1N1) | 1 | D | 1/23/07 | 8 | 13 | M | Florence |
| ABW40223 | A/Kentucky/UR06-0058/2007(H1N1) |  | A | 1/23/07 | 8 | 10 | F | Florence |
| ABV29611 | A/Kentucky/UR06-0059/2007(H1N1) |  | D | 1/23/07 | 8 | 14 | F | Florence |
| ABW40135 | A/Kentucky/UR06-0061/2007(H1N1) |  | A | 1/22/07 | 8 | 6 | M | Florence |
| ABV29875 | A/Kentucky/UR06-0062/2007(H1N1) |  | A | 1/23/07 | 8 | 8 | F | Florence |
| ABV30502 | A/Kentucky/UR06-0069/2007(H1N1) | 2 | A | 1/24/07 | 8 | 9 | F | Florence |
| ABW40234 | A/Kentucky/UR06-0071/2007(H1N1) | 2 | A | 1/24/07 | 8 | 6 | F | Florence |
| ABV29842 | A/Kentucky/UR06-0072/2007(H1N1) | 2 | A | 1/24/07 | 8 | 12 | F | Florence |
| ABW71326 | A/Kentucky/UR06-0081/2007(H1N1) | 1,2 | A | 1/25/07 | 8 | 6 | F | Florence |
| ABV29677 | A/Kentucky/UR06-0082/2007(H1N1) |  | A | 1/26/07 | 8 | 8 | M | Florence |
| ABV29952 | A/Kentucky/UR06-0097/2007(H1N1) |  | D | 1/29/07 | 9 | 6 | M | Florence |
| ABW40322 | A/Kentucky/UR06-0123/2007(H1N1) |  | A | 1/30/07 | 9 | 12 | M | Hopkinsville |
| ABW40311 | A/Kentucky/UR06-0127/2007(H1N1) |  | A | 1/31/07 | 9 | 3 | F | Hopkinsville |
| ABV30590 | A/Kentucky/UR06-0128/2007(H1N1) | 1 | A | 1/31/07 | 9 | 1 | M | Hopkinsville |
| ABV29886 | A/Kentucky/UR06-0129/2007(H1N1) | 2 | A | 1/30/07 | 9 | 6 | M | Florence |
| ABW40564 | A/Kentucky/UR06-0154/2007(H1N1) | 2 | A | 2/2/07 | 9 | 3 | M | Hopkinsville |
| ABW40465 | A/Kentucky/UR06-0161/2007(H1N1) | 1 | C | 2/5/07 | 10 | 17 | M | Hopkinsville |
| ABX58447 | A/Kentucky/UR06-0162/2007(H1N1) | 2 | A | 2/5/07 | 10 | 3 | M | Hopkinsville |
| ABY51115 | A/Kentucky/UR06-0181/2007(H1N1) | 2 | A | 2/6/07 | 10 | 2 | M | Hopkinsville |
| ABX58623 | A/Kentucky/UR06-0182/2007(H1N1) |  | C | 2/6/07 | 10 | 7 | F | Hopkinsville |
| ABW40520 | A/Kentucky/UR06-0183/2007(H1N1) |  | B | 2/6/07 | 10 | 7 | F | Hopkinsville |
| ABW86518 | A/Kentucky/UR06-0184/2007(H1N1) |  | A | 2/6/07 | 10 | 3 | F | Hopkinsville |
| ABV45925 | A/Kentucky/UR06-0187/2007(H1N1) |  | A | 2/5/07 | 10 | 4 | M | Florence |
| ABV30117 | A/Kentucky/UR06-0188/2007(H1N1) |  | A | 2/5/07 | 10 | 8 | F | Florence |
| ABW86353 | A/Kentucky/UR06-0220/2007(H1N1) | 1 | A | 2/8/07 | 10 | 6 | F | Florence |
| ABV30172 | A/Kentucky/UR06-0240/2007(H1N1) | 2 | A | 2/12/07 | 11 | 4 | M | Florence |
| ABV30546 | A/Kentucky/UR06-0257/2007(H1N1) | 2 | A | 2/13/07 | 11 | 10 | M | Hopkinsville |
| ABX58645 | A/Kentucky/UR06-0258/2007(H1N1) | 2 | A | 2/13/07 | 11 | 5 | F | Hopkinsville |
| ABX58304 | A/Kentucky/UR06-0259/2007(H1N1) | 2 | A | 2/13/07 | 11 | 6 | M | Hopkinsville |
| ABW40509 | A/Kentucky/UR06-0327/2007(H1N1) |  | A | 2/16/07 | 11 | 9 | M | Hopkinsville |
| ABW40476 | A/Kentucky/UR06-0328/2007(H1N1) | 1 | A | 2/16/07 | 11 | 5 | F | Hopkinsville |
| ABW91536 | A/Kentucky/UR06-0339/2007(H1N1) |  | A | 2/19/07 | 12 | 6 | F | Hopkinsville |
| ABV29743 | A/Kentucky/UR06-0363/2007(H1N1) | 1 | D | 2/20/07 | 12 | 2 | M | Florence |
| ABW71480 | A/Kentucky/UR06-0371/2007(H1N1) |  | A | 2/21/07 | 12 | 8 | M | Hopkinsville |
| ABX58535 | A/Kentucky/UR06-0391/2007(H1N1) |  | B | 2/22/07 | 12 | 6 | M | Hopkinsville |
| ABX58480 | A/Kentucky/UR06-0401/2007(H1N1) | 2 | A | 2/23/07 | 12 | 1 | M | Hopkinsville |
| ABW86616 | A/Kentucky/UR06-0424/2007(H1N1) |  | C | 2/26/07 | 13 | 1 | M | Hopkinsville |
| ABX58491 | A/Kentucky/UR06-0425/2007(H1N1) | 1 | B | 2/26/07 | 13 | 8 | M | Hopkinsville |
| ABW91393 | A/Kentucky/UR06-0449/2007(H1N1) |  | C | 2/27/07 | 13 | 7 | F | Hopkinsville |
| ABW39926 | A/Kentucky/UR06-0476/2007(H1N1) | 1 | F | 3/1/07 | 13 | 7 | M | Florence |
| ABV45958 | A/Kentucky/UR06-0538/2007(H1N1) |  | C | 3/7/07 | 14 | 7 | M | Hopkinsville |
| ABV30535 | A/Kentucky/UR06-0539/2007(H1N1) |  | C | 3/7/07 | 14 | 10 | M | Hopkinsville |
| ABV30161 | A/Kentucky/UR06-0553/2007(H1N1) |  | C | 3/8/07 | 14 | 15 | M | Hopkinsville |
| ABV29556 | A/Michigan/UR06-0015/2006(H1N1) | 1 | C | 12/28/06 | 4 | 10 | M | Royal Oak |
| ABX58249 | A/Mississippi/UR06-0014/2006(H1N1) | 1,2 | A | 12/28/06 | 4 | 27 | M | Aberdeen |
| ABW71370 | A/Mississippi/UR06-0047/2007(H1N1) | 1 | B | 1/22/07 | 8 | 8 | M | Aberdeen |
| ABV30348 | A/Mississippi/UR06-0048/2007(H1N1) | 1 | A | 1/22/07 | 8 | 48 | F | Aberdeen |
| ABV30106 | A/Mississippi/UR06-0086/2007(H1N1) |  | A | 1/26/07 | 8 | 9 | M | Aberdeen |
| ABV30007 | A/Mississippi/UR06-0130/2007(H1N1) |  | A | 1/31/07 | 9 | 67 | F | Aberdeen |
| ABV30095 | A/Mississippi/UR06-0142/2007(H1N1) |  | A | 2/1/07 | 9 | 6 | M | Aberdeen |
| ABV30040 | A/Mississippi/UR06-0145/2007(H1N1) | 1 | A | 2/2/07 | 9 | 26 | M | Aberdeen |
| ABV29688 | A/Mississippi/UR06-0242/2007(H1N1) |  | A | 2/12/07 | 11 | 15 | F | Aberdeen |
| ABW36266 | A/Mississippi/UR06-0378/2007(H1N1) | 1 | F | 2/20/07 | 12 | 28 | M | Aberdeen |
| ABV30469 | A/Mississippi/UR06-0537/2007(H1N1) |  | A | 3/7/07 | 14 | 8 | F | Aberdeen |
| ABW36299 | A/Mississippi/UR06-0595/2007(H1N1) | 2 | A | 3/13/07 | 15 | 3 | M | Aberdeen |
| ABW36277 | A/New York/UR06-0056/2007(H1N1) | 2 | A | 1/23/07 | 8 | 5 | F | Bronx |
| ABW40124 | A/New York/UR06-0134/2007(H1N1) | 1,2 | A | 1/31/07 | 9 | 7 | F | Bronx |
| ABW39981 | A/New York/UR06-0199/2007(H1N1) | 1 | H | 2/7/07 | 10 | 5 | M | Bronx |
| ABW40003 | A/New York/UR06-0253/2007(H1N1) | 1 | C | 2/13/07 | 11 | 59 | F | Bronx |
| ABW91294 | A/New York/UR06-0326/2007(H1N1) | 1 | H | 2/16/07 | 11 | 54 | F | Bronx |
| ABV29644 | A/New York/UR06-0386/2007(H1N1) | 1 | C | 2/22/07 | 12 | 46 | F | Bronx |
| ABW71304 | A/North Carolina/UR06-0011/2006(H1N1) | 1 | C | 12/18/06 | 3 | 4 | F | Winston-Salem |
| ABW40300 | A/North Carolina/UR06-0099/2007(H1N1) | 1 | C | 1/29/07 | 9 | 59 | M | Graham |
| ABW40487 | A/North Carolina/UR06-0364/2007(H1N1) | 1,2 | A | 2/19/07 | 12 | 5 | F | Graham |
| ABY51159 | A/North Carolina/UR06-0365/2007(H1N1) | 2 | A | 2/19/07 | 12 | 7 | M | Graham |
| ABW40432 | A/Ohio/UR06-0091/2007(H1N1) |  | A | 1/29/07 | 9 | 9 | M | Fairfield |
| ABV30623 | A/Ohio/UR06-0100/2007(H1N1) |  | C | 1/29/07 | 9 | 8 | M | Washington |
| ABW91514 | A/Ohio/UR06-0112/2007(H1N1) |  | C | 1/30/07 | 9 | 16 | M | Oberlin |
| ABW86496 | A/Ohio/UR06-0121/2007(H1N1) | 2 | A | 1/30/07 | 9 | 11 | M | Washington |
| ABV30634 | A/Ohio/UR06-0122/2007(H1N1) | 2 | A | 1/30/07 | 9 | 1 | F | Washington |
| ABW86441 | A/Ohio/UR06-0166/2007(H1N1) | 2 | A | 2/5/07 | 10 | 6 | M | Washington |
| ABW40685 | A/Ohio/UR06-0177/2007(H1N1) | 1 | C | 2/6/07 | 10 | 9 | F | Washington |
| ABW86419 | A/Ohio/UR06-0233/2007(H1N1) | 2 | A | 2/9/07 | 10 | 7 | F | Fairfield |
| ABV30579 | A/Ohio/UR06-0325/2007(H1N1) |  | A | 2/16/07 | 11 | 4 | F | Fairfield |
| ABW40289 | A/Ohio/UR06-0341/2007(H1N1) |  | A | 2/19/07 | 12 | 49 | F | Fairfield |
| ABX58590 | A/Ohio/UR06-0353/2007(H1N1) | 2 | A | 2/19/07 | 12 | 4 | F | Washington |
| ABW86529 | A/Ohio/UR06-0394/2007(H1N1) |  | C | 2/23/07 | 12 | 13 | M | Toledo |
| ABX58403 | A/Ohio/UR06-0411/2007(H1N1) |  | A | 2/26/07 | 13 | 9 | F | North Canton |
| ABY51181 | A/Ohio/UR06-0429/2007(H1N1) | 1 | C | 2/23/07 | 12 | 11 | M | Washington |
| ABW40586 | A/Ohio/UR06-0443/2007(H1N1) | 1,2 | A | 2/26/07 | 13 | 7 | F | Washington |
| ABW40498 | A/Ohio/UR06-0465/2007(H1N1) | 2 | A | 2/28/07 | 13 | 7 | M | Washington |
| ABW40421 | A/Ohio/UR06-0518/2007(H1N1) | 2 | A | 3/5/07 | 14 | 10 | F | Oberlin |
| ABW71447 | A/Ohio/UR06-0521/2007(H1N1) | 2 | A | 3/5/07 | 14 | 1 | M | Washington |
| ABX58458 | A/Ohio/UR06-0522/2007(H1N1) | 1 | A | 3/6/07 | 14 | 4 | M | Washington |
| ABW91525 | A/Oklahoma/UR06-0063/2007(H1N1) | 1 | B | 1/22/07 | 8 | 5 | M | Choctaw |
| ABX58370 | A/Oklahoma/UR06-0241/2007(H1N1) | 2 | A | 1/22/07 | 8 | 24 | M | Norman |
| ABX58601 | A/Oklahoma/UR06-0519/2007(H1N1) | 2 | A | 3/5/07 | 14 | 41 | F | Norman |
| ABW91415 | A/Oregon/UR06-0179/2007(H1N1) |  | B | 2/5/07 | 10 | 10 | F | Newberg |
| ABX58359 | A/Oregon/UR06-0185/2007(H1N1) |  | B | 2/5/07 | 10 | 13 | F | Newberg |
| ABX58315 | A/Oregon/UR06-0186/2007(H1N1) | 1 | B | 2/5/07 | 10 | 9 | F | Newberg |
| ABX58711 | A/Oregon/UR06-0219/2007(H1N1) |  | B | 2/7/07 | 10 | 10 | F | Newberg |
| ABW40608 | A/Oregon/UR06-0230/2007(H1N1) |  | B | 2/9/07 | 10 | 11 | M | Newberg |
| ABW40355 | A/Oregon/UR06-0231/2007(H1N1) |  | B | 2/8/07 | 10 | 13 | F | Newberg |
| ABW40080 | A/Tennessee/UR06-0045/2007(H1N1) | 1 | F | 1/18/07 | 7 | 2 | F | Dyersburg |
| ABX58568 | A/Tennessee/UR06-0076/2007(H1N1) |  | A | 1/25/07 | 8 | 3 | M | Tullahoma |
| ABW40344 | A/Tennessee/UR06-0078/2007(H1N1) |  | B | 1/26/07 | 8 | 6 | F | Tullahoma |
| ABX58524 | A/Tennessee/UR06-0080/2007(H1N1) | 1 | B | 1/26/07 | 8 | 2 | M | Tullahoma |
| ABW39816 | A/Tennessee/UR06-0087/2007(H1N1) | 2 | A | 1/26/07 | 8 | 6 | M | Dyersburg |
| ABW91459 | A/Tennessee/UR06-0113/2007(H1N1) |  | B | 1/29/07 | 9 | 5 | F | Tullahoma |
| ABW86408 | A/Tennessee/UR06-0119/2007(H1N1) | 1 | B | 1/29/07 | 9 | 8 | F | Tullahoma |
| ABW40674 | A/Tennessee/UR06-0120/2007(H1N1) |  | B | 1/29/07 | 9 | 14 | F | Tullahoma |
| ABW71425 | A/Tennessee/UR06-0124/2007(H1N1) |  | C | 1/29/07 | 9 | 9 | F | Tullahoma |
| ABW40575 | A/Tennessee/UR06-0151/2007(H1N1) | 1,2 | A | 2/2/07 | 9 | 3 | M | Knoxville |
| ABW40652 | A/Tennessee/UR06-0152/2007(H1N1) | 2 | A | 2/2/07 | 9 | 8 | F | Knoxville |
| ABW86540 | A/Tennessee/UR06-0234/2007(H1N1) | 2 | A | 2/12/07 | 11 | 13 | F | Knoxville |
| ABX58502 | A/Tennessee/UR06-0238/2007(H1N1) |  | A | 2/12/07 | 11 | 4 | M | Knoxville |
| ABW71458 | A/Tennessee/UR06-0239/2007(H1N1) |  | A | 2/12/07 | 11 | 4 | M | Knoxville |
| ABW86463 | A/Tennessee/UR06-0262/2007(H1N1) | 1 | C | 2/13/07 | 11 | 3 | M | Tullahoma |
| ABY51170 | A/Tennessee/UR06-0277/2007(H1N1) | 1 | B | 2/14/07 | 11 | 5 | M | Tullahoma |
| ABW71436 | A/Tennessee/UR06-0294/2007(H1N1) |  | A | 2/14/07 | 11 | 2 | M | Knoxville |
| ABX58425 | A/Tennessee/UR06-0312/2007(H1N1) |  | B | 2/15/07 | 11 | 2 | M | Tullahoma |
| ABW91338 | A/Tennessee/UR06-0388/2007(H1N1) |  | A | 2/22/07 | 12 | 5 | M | Knoxville |
| ABW86507 | A/Tennessee/UR06-0414/2007(H1N1) |  | B | 2/26/07 | 13 | 3.5 | F | Knoxville |
| ABW91492 | A/Tennessee/UR06-0459/2007(H1N1) |  | B | 2/27/07 | 13 | 3 | F | Tullahoma |
| ABW86562 | A/Tennessee/UR06-0473/2007(H1N1) | 2 | A | 3/1/07 | 13 | 8 | M | Knoxville |
| ABW91470 | A/Tennessee/UR06-0508/2007(H1N1) |  | B | 3/5/07 | 14 | 4 | M | Knoxville |
| ABX58579 | A/Tennessee/UR06-0509/2007(H1N1) | 1,2 | A | 3/5/07 | 14 | 12 | F | Knoxville |
| ABX58546 | A/Tennessee/UR06-0523/2007(H1N1) |  | B | 3/5/07 | 14 | 14 | F | Lebanon |
| ABV29545 | A/Texas/UR06-0012/2006(H1N1) | 1 | B | 12/20/06 | 3 | 3.5 | M | Conroe |
| ABV29754 | A/Texas/UR06-0025/2007(H1N1) | 1,2 | A | 1/11/07 | 6 | 7 | F | Conroe |
| ABY51071 | A/Texas/UR06-0026/2007(H1N1) | 1 | E | 1/11/07 | 6 | 1 | F | Conroe |
| ABW91580 | A/Texas/UR06-0039/2007(H1N1) | 1,2 | A | 1/16/07 | 7 | 9 | M | Conroe |
| ABW91371 | A/Texas/UR06-0133/2007(H1N1) | 1 | D | 1/31/07 | 9 | 9 | M | Conroe |
| ABW40443 | A/Texas/UR06-0157/2007(H1N1) | 1 | B | 2/5/07 | 10 | 5 | F | Conroe |
| ABV30557 | A/Texas/UR06-0174/2007(H1N1) |  | D | 2/6/07 | 10 | 6 | M | Conroe |
| ABW36255 | A/Texas/UR06-0175/2007(H1N1) |  | A | 2/6/07 | 10 | 8 | F | Conroe |
| ABW71337 | A/Texas/UR06-0176/2007(H1N1) |  | A | 2/6/07 | 10 | 6 | M | Conroe |
| ABV30183 | A/Texas/UR06-0193/2007(H1N1) |  | A | 2/6/07 | 10 | 8 | M | Conroe |
| ABV45936 | A/Texas/UR06-0195/2007(H1N1) | 1 | D | 2/6/07 | 10 | 4 | F | Conroe |
| ABV29974 | A/Texas/UR06-0196/2007(H1N1) |  | A | 2/6/07 | 10 | 9 | M | Conroe |
| ABV29798 | A/Texas/UR06-0203/2007(H1N1) | 2 | A | 2/7/07 | 10 | 4 | M | Conroe |
| ABW91228 | A/Texas/UR06-0204/2007(H1N1) | 1,2 | A | 2/7/07 | 10 | 4 | F | Conroe |
| ABW91316 | A/Texas/UR06-0216/2007(H1N1) | 2 | A | 2/8/07 | 10 | 5 | M | Conroe |
| ABY51060 | A/Texas/UR06-0217/2007(H1N1) | 1 | F | 2/8/07 | 10 | 16 | M | Conroe |
| ABW36310 | A/Texas/UR06-0270/2007(H1N1) |  | D | 2/13/07 | 11 | 5 | M | Conroe |
| ABW36233 | A/Texas/UR06-0271/2007(H1N1) | 2 | A | 2/13/07 | 11 | 6 | F | Conroe |
| ABV45903 | A/Texas/UR06-0303/2007(H1N1) |  | D | 2/15/07 | 11 | 5 | F | Conroe |
| ABW71315 | A/Texas/UR06-0305/2007(H1N1) |  | B | 2/15/07 | 11 | 13 | M | Conroe |
| ABV82561 | A/Texas/UR06-0306/2007(H1N1) | 1 | D | 2/15/07 | 11 | 18 | F | Conroe |
| ABW39992 | A/Texas/UR06-0308/2007(H1N1) | 2 | A | 2/15/07 | 11 | 4 | M | Conroe |
| ABW39937 | A/Texas/UR06-0309/2007(H1N1) |  | A | 2/15/07 | 11 | 6 | M | Conroe |
| ABW86342 | A/Texas/UR06-0342/2007(H1N1) |  | D | 2/19/07 | 12 | 5 | F | Conroe |
| ABW86364 | A/Texas/UR06-0357/2007(H1N1) |  | A | 2/20/07 | 12 | 14 | M | Conroe |
| ABV30194 | A/Texas/UR06-0359/2007(H1N1) |  | A | 2/20/07 | 12 | 3 | F | Conroe |
| ABV29655 | A/Texas/UR06-0380/2007(H1N1) | 1,2 | A | 2/21/07 | 12 | 7 | F | Conroe |
| ABW39893 | A/Texas/UR06-0397/2007(H1N1) | 2 | A | 2/23/07 | 12 | 9 | M | Conroe |
| ABV29787 | A/Texas/UR06-0398/2007(H1N1) | 1 | D | 2/22/07 | 12 | 1 | M | Conroe |
| ABX58722 | A/Texas/UR06-0420/2007(H1N1) | 1 | E | 2/26/07 | 13 | 8 | M | Conroe |
| ABW36288 | A/Texas/UR06-0444/2007(H1N1) | 2 | A | 2/27/07 | 13 | 3 | M | Conroe |
| ABW91283 | A/Texas/UR06-0445/2007(H1N1) | 2 | A | 2/27/07 | 13 | 1 | F | Conroe |
| ABV30018 | A/Texas/UR06-0461/2007(H1N1) |  | A | 2/27/07 | 13 | 9 | F | Conroe |
| ABV29864 | A/Texas/UR06-0467/2007(H1N1) | 1 | E | 3/1/07 | 13 | 5 | M | Conroe |
| ABW39787 | A/Texas/UR06-0468/2007(H1N1) | 1 | E | 3/1/07 | 13 | 6 | M | Conroe |
| ABV29622 | A/Texas/UR06-0502/2007(H1N1) |  | A | 3/5/07 | 14 | 6 | F | Conroe |
| ABX58260 | A/Texas/UR06-0503/2007(H1N1) | 1 | E | 3/5/07 | 14 | 5 | M | Conroe |
| ABV30051 | A/Texas/UR06-0526/2007(H1N1) |  | C | 3/6/07 | 14 | 7 | F | Conroe |
| ABW36211 | A/Texas/UR06-0540/2007(H1N1) |  | A | 3/6/07 | 14 | 5 | F | Conroe |
| ABW36222 | A/Texas/UR06-0542/2007(H1N1) | 1 | E | 3/7/07 | 14 | 6 | M | Conroe |
| ABV29996 | A/Texas/UR06-0563/2007(H1N1) | 1 | A | 3/7/07 | 14 | 3 | F | Conroe |
| ABW40102 | A/Texas/UR06-0582/2007(H1N1) | 1,2 | A | 3/12/07 | 15 | 6 | M | Conroe |
| ABW40058 | A/Vermont/UR06-0035/2007(H1N1) | 1,2 | A | 1/16/07 | 7 | 2 | M | Bennington |
| ABV29666 | A/Vermont/UR06-0050/2007(H1N1) | 1,2 | A | 1/22/07 | 8 | 6 | M | Bennington |
| ABV30359 | A/Vermont/UR06-0051/2007(H1N1) | 2 | A | 1/22/07 | 8 | 3 | M | Bennington |
| ABV30337 | A/Vermont/UR06-0089/2007(H1N1) |  | B | 1/26/07 | 8 | 6 | F | Bennington |
| ABV30139 | A/Vermont/UR06-0090/2007(H1N1) |  | A | 1/26/07 | 8 | 7 | F | Bennington |
| ABV30150 | A/Vermont/UR06-0301/2007(H1N1) | 1 | B | 2/15/07 | 11 | 3 | F | Bennington |
| ABW71348 | A/Vermont/UR06-0472/2007(H1N1) |  | A | 3/1/07 | 13 | 2 | F | Bennington |
| ABV30293 | A/Vermont/UR06-0485/2007(H1N1) | 1 | A | 3/2/07 | 13 | 1 | M | Bennington |
| ABW39970 | A/Vermont/UR06-0511/2007(H1N1) | 1 | A | 3/5/07 | 14 | 9 | M | Bennington |
| ABW40157 | A/Vermont/UR06-0556/2007(H1N1) | 2 | A | 3/8/07 | 14 | 3 | F | Bennington |
| ABW39904 | A/Vermont/UR06-0573/2007(H1N1) | 2 | A | 3/12/07 | 15 | 3 | M | Bennington |
| ABV30370 | A/Vermont/UR06-0574/2007(H1N1) | 2 | A | 3/11/07 | 15 | 4 | M | Bennington |
| ABV45881 | A/Vermont/UR06-0575/2007(H1N1) | 2 | A | 3/12/07 | 15 | 3 | F | Bennington |
| ABV29897 | A/Vermont/UR06-0576/2007(H1N1) | 1 | A | 3/12/07 | 15 | 3 | M | Bennington |
| ABW91602 | A/Virginia/UR06-0075/2007(H1N1) | 1 | A | 1/25/07 | 8 | 5 | M | Weber City |
| ABX58414 | A/Virginia/UR06-0092/2007(H1N1) |  | A | 1/29/07 | 9 | 3 | M | Richmond |
| ABV30612 | A/Virginia/UR06-0109/2007(H1N1) |  | A | 1/29/07 | 9 | 4 | F | Weber City |
| ABW91503 | A/Virginia/UR06-0114/2007(H1N1) | 2 | A | 1/30/07 | 9 | 5 | F | Weber City |
| ABW71414 | A/Virginia/UR06-0117/2007(H1N1) |  | C | 1/30/07 | 9 | 8 | F | Richmond |
| ABW40267 | A/Virginia/UR06-0139/2007(H1N1) | 2 | A | 2/1/07 | 9 | 1 | F | Weber City |
| ABW40410 | A/Virginia/UR06-0164/2007(H1N1) | 2 | A | 2/5/07 | 10 | 4 | F | Weber City |
|  | A/Virginia/UR06-0228/2007(H1N1) | 2 | A | 2/9/07 | 10 | 10 | M | Richmond |
| ABW40531 | A/Virginia/UR06-0244/2007(H1N1) | 1 | A | 2/12/07 | 11 | 6 | F | Richmond |
| ABV30601 | A/Virginia/UR06-0245/2007(H1N1) |  | A | 2/12/07 | 11 | 13 | F | Richmond |
| ABW40366 | A/Virginia/UR06-0254/2007(H1N1) | 1 | C | 2/13/07 | 11 | 8 | M | Weber City |
| ABX58381 | A/Virginia/UR06-0266/2007(H1N1) |  | A | 2/13/07 | 11 | 9 | M | Richmond |
| ABW40399 | A/Virginia/UR06-0267/2007(H1N1) |  | A | 2/13/07 | 11 | 13 | M | Richmond |
| ABX58337 | A/Virginia/UR06-0295/2007(H1N1) |  | C | 2/14/07 | 11 | 42 | M | Weber City |
| ABW91349 | A/Virginia/UR06-0297/2007(H1N1) | 2 | A | 2/14/07 | 11 | 7 | F | Richmond |
| ABY51148 | A/Virginia/UR06-0332/2007(H1N1) | 1 | B | 2/19/07 | 12 | 11 | M | Weber City |
| ABY51192 | A/Virginia/UR06-0346/2007(H1N1) | 2 | A | 2/19/07 | 12 | 6 | M | Richmond |
| ABX58634 | A/Virginia/UR06-0351/2007(H1N1) | 2 | A | 2/20/07 | 12 | 7 | M | Richmond |
| ABW91646 | A/Virginia/UR06-0360/2007(H1N1) | 2 | A | 2/20/07 | 12 | 7 | M | Richmond |
| ABW40377 | A/Virginia/UR06-0384/2007(H1N1) | 1 | A | 2/21/07 | 12 | 4 | M | Richmond |
| ABX58557 | A/Virginia/UR06-0387/2007(H1N1) | 1 | A | 2/22/07 | 12 | 37 | F | Weber City |
| ABW91437 | A/Virginia/UR06-0549/2007(H1N1) |  | C | 3/7/07 | 14 | 28 | M | Weber City |
| ABW91360 | A/Virginia/UR06-0562/2007(H1N1) | 1 | A | 3/8/07 | 14 | 4 | M | Richmond |
| ABX58348 | A/Virginia/UR06-0594/2007(H1N1) | 1,2 | A | 3/13/07 | 15 | 9 | F | Richmond |
