# Supplementary material for: Molecular Epidemiology of A/H3N2 and A/H1N1 Influenza Virus during a Single Epidemic Season in the United States
Source: PLoS Pathog. 2008 Aug 22;4(8):e1000133. doi: 10.1371/journal.ppat.1000133 (PMC2495036; doi:10.1371/journal.ppat.1000133)
Supplement: Table S3 — Influenza A viruses used in Figures 1– 5, and S1, S2, S3, S4, S5, S6. GenBank accession numbers, collection dates, and age and sex of patient from whom isolate were assembled for 48 A/H1N1 influenza viruses sampled globally from 2001–2006. GenBank accession numbers from the Influenza Virus Resource refer to the PB2 gene segment (http://www.ncbi.nlm.nih.gov/genomes/FLU/FLU.html). (0.09 MB DOC) [file ppat.1000133.s013.doc]

**Table S3**. Influenza A viruses used in Figures 1-5, and S1-S6. GenBank accession numbers, collection dates, and age and sex of patient from whom isolate were assembled for 48 A/H1N1 influenza viruses sampled globally from 2001 – 2006. GenBank accession numbers from the Influenza Virus Resource refer to the PB2 gene segment (<http://www.ncbi.nlm.nih.gov/genomes/FLU/FLU.html>).

| **Accession** | **Collection Date (M/D/Y)** | **Isolate name** | **Patient Age (years)** | **Patient Sex** |
| --- | --- | --- | --- | --- |
| ABR28910 | 7/1/05 | A/Auckland/619/2005(H1N1) | 46 | M |
| ABE11689 | 1/9/01 | A/Canterbury/01/2001(H1N1) | 24 | F |
| ABC42760 | 9/12/04 | A/Canterbury/106/2004(H1N1) | 13 | F |
| ABD95272 | 8/2/01 | A/Canterbury/119/2001(H1N1) | 20 | F |
| ABD95217 | 7/9/01 | A/Canterbury/53/2001(H1N1) | 27 | F |
| ABN51098 | 2003 | A/Memphis/5/2003(H1N1) | n/a | n/a |
| ABO32958 | 2001 | A/Memphis/6/2001(H1N1) | n/a | n/a |
| AAZ83263 | 12/26/02 | A/New York/220/2002(H1N1) | 33 | F |
| ABA42257 | 3/21/03 | A/New York/221/2003(H1N1) | <1 | F |
| ABA12706 | 1/12/03 | A/New York/222/2003(H1N1) | 3 | M |
| ABA12720 | 1/15/03 | A/New York/223/2003(H1N1) | 5 | M |
| AAZ83987 | 3/25/03 | A/New York/227/2003(H1N1) | 4 | M |
| ABA87067 | 1/14/03 | A/New York/228/2003(H1N1) | 30 | M |
| ABA08485 | 1/27/03 | A/New York/230/2003(H1N1) | 20 | M |
| ABA87090 | 12/21/02 | A/New York/291/2002(H1N1) | <1 | M |
| ABB02802 | 3/5/03 | A/New York/292/2003(H1N1) | <1 | F |
| ABB02813 | 1/28/03 | A/New York/293/2003(H1N1) | 6 | M |
| ABA12739 | 2/28/03 | A/New York/348/2003(H1N1) | <1 | F |
| ABB80113 | 3/4/03 | A/New York/350/2003(H1N1) | <1 | M |
| ABA18155 | 1/22/03 | A/New York/399/2003(H1N1) | 49 | M |
| ABB02934 | 1/22/01 | A/New York/443/2001(H1N1) | <1 | M |
| ABD15525 | 1/14/03 | A/New York/483/2003(H1N1) | 10 | M |
| ABD60789 | 1/20/03 | A/New York/484/2003(H1N1) | 8 | M |
| ABB03133 | 1/21/03 | A/New York/486/2003(H1N1) | 50 | M |
| ABC41724 | 1/23/03 | A/New York/488/2003(H1N1) | 1 | M |
| ABB82215 | 2/12/03 | A/New York/493/2003(H1N1) | 43 | F |
| ABB82226 | 12/5/02 | A/New York/494/2002(H1N1) | 3 | M |
| ABB03155 | 2/25/03 | A/New York/496/2003(H1N1) | 2 | F |
| ABB53750 | 2/26/03 | A/New York/497/2003(H1N1) | 1 | F |
| ABK79969 | 4/10/06 | A/New York/8/2006(H1N1) | 62 | F |
| ABI21221 | 9/7/05 | A/Otago/5/2005(H1N1) | 23 | F |
| ABP49403 | 2005 | A/South Australia/51/2005(H1N1) | n/a | n/a |
| ABJ16663 | 2005 | A/South Australia/55/2005(H1N1) | n/a | n/a |
| ABJ16674 | 2005 | A/South Australia/56/2005(H1N1) | n/a | n/a |
| ABJ16685 | 2005 | A/South Australia/57/2005(H1N1) | n/a | n/a |
| ABJ16696 | 2005 | A/South Australia/58/2005(H1N1) | n/a | n/a |
| ABJ09194 | 8/31/05 | A/Waikato/11/2005(H1N1) | 6 | M |
| ABI21232 | 9/10/05 | A/Waikato/13/2005(H1N1) | 17 | F |
| ABI22158 | 9/19/05 | A/Waikato/14/2005(H1N1) | 52 | M |
| ABO32688 | 8/12/05 | A/Waikato/17/2005(H1N1) | 35 | F |
| ABK40699 | 8/8/05 | A/Waikato/4/2005(H1N1) | 9 | M |
| ABF82829 | 5/10/01 | A/Wellington/1/2001(H1N1) | 29 | M |
| ABI21199 | 9/6/05 | A/Wellington/10/2005(H1N1) | 9 | F |
| ABI30575 | 9/8/05 | A/Wellington/11/2005(H1N1) | 9 | F |
| ABI21210 | 9/13/05 | A/Wellington/12/2005(H1N1) | 30 | M |
| ABI21243 | 9/20/05 | A/Wellington/13/2005(H1N1) | 8 | F |
| ABI92389 | 10/5/05 | A/Wellington/14/2005(H1N1) | 43 | M |
| ABO32980 | 8/9/05 | A/Western Australia/77/2005(H1N1) | n/a | n/a |
