# Supplementary material for: Molecular Epidemiology of A/H3N2 and A/H1N1 Influenza Virus during a Single Epidemic Season in the United States
Source: PLoS Pathog. 2008 Aug 22;4(8):e1000133. doi: 10.1371/journal.ppat.1000133 (PMC2495036; doi:10.1371/journal.ppat.1000133)
Supplement: Table S4 — Influenza A viruses used in Figure 1. GenBank accession numbers and collection dates for the HA gene segment of 21 A/H1N1 influenza viruses sampled globally from 2006, including the A/H1N1 components of the influenza vaccines for 2006–2007 (A/New Caledonia/20.1999), 2007–2008 (A/Solomon Islands/3/2006), and 2008–2009 (A/Brisbane/59/2007). GenBank accession numbers from the Influenza Virus Resource refer to the PB2 gene segment (http://www.ncbi.nlm.nih.gov/genomes/FLU/FLU.html), and accession numbers starting with ISD are from the Los Alamos National Laboratory's Influenza Sequence Database (www.flu.lanl.gov). (0.05 MB DOC) [file ppat.1000133.s014.doc]

**Table S4**. Influenza A viruses used in Figure 1. GenBank accession numbers and collection dates for the HA gene segment of 21 A/H1N1 influenza viruses sampled globally from 2006, including the A/H1N1 components of the influenza vaccines for 2006-2007 (A/New Caledonia/20.1999), 2007-2008 (A/Solomon Islands/3/2006), and 2008-2009 (A/Brisbane/59/2007). GenBank accession numbers from the Influenza Virus Resource refer to the PB2 gene segment (<http://www.ncbi.nlm.nih.gov/genomes/FLU/FLU.html>), and accession numbers starting with ISD are from the Los Alamos National Laboratory’s Influenza Sequence Database ([www.flu.lanl.gov](http://www.flu.lanl.gov/)).

| **Accession** | **Collection Date/Year** | **Isolate name** |
| --- | --- | --- |
| ABV45654 | 2006 | A/Hong Kong/948/2006(H1N1) |
| CAC86622 | 1999 | A/New Caledonia/20/1999(H1N1) |
| ABU99109 | 8/21/06 | A/Solomon Islands/3/2006(H1N1) |
| ABU50587 | 2006 | A/St. Petersburg/08/2006 X-163(H1N1) |
| ACA28844 | 7/01/07 | A/Brisbane/59/2007(H1N1) |
| ABU50588 | 2006 | A/St. Petersburg/08/2006 X-163A(H1N1) |
| ABU50589 | 2006 | A/St. Petersburg/08/2006 X-163B(H1N1) |
| ISDN214690 | 2006 | A/Stockholm/15/06 (H1N1) |
| ISDN214693 | 2006 | A/Stockholm/17/06 (H1N1) |
| ISDN229826 | 2006 | A/Stockholm/23/06 (H1N1) |
| ISDN214687 | 2006 | A/Stockholm/4/06 (H1N1) |
| ABS71673 | 2006 | A/Thailand/CU32/2006(H1N1) |
| ABS71664 | 2006 | A/Thailand/CU41/2006(H1N1) |
| ABS71670 | 2006 | A/Thailand/CU44/2006(H1N1) |
| ABS71668 | 2006 | A/Thailand/CU51/2006(H1N1) |
| ABS71665 | 2006 | A/Thailand/CU53/2006(H1N1) |
| ABS71669 | 2006 | A/Thailand/CU57/2006(H1N1) |
| ABS71666 | 2006 | A/Thailand/CU67/2006(H1N1) |
| ABS71671 | 2006 | A/Thailand/CU68/2006(H1N1) |
| ABS71672 | 2006 | A/Thailand/CU75/2006(H1N1) |
| ABS71667 | 2006 | A/Thailand/CU88/2006(H1N1) |
