# Supplementary material for: Molecular Epidemiology of A/H3N2 and A/H1N1 Influenza Virus during a Single Epidemic Season in the United States
Source: PLoS Pathog. 2008 Aug 22;4(8):e1000133. doi: 10.1371/journal.ppat.1000133 (PMC2495036; doi:10.1371/journal.ppat.1000133)
Supplement: Table S5 — Influenza A viruses used in Figure S4. GenBank accession numbers and collection dates for the NA gene of 17 A/H1N1 influenza viruses sampled globally from 2006, including the A/H1N1 components of the influenza vaccines for 2006–2007 (A/New Caledonia/20/1999) and 2007–2008 (A/Solomon Islands/3/2006). GenBank accession numbers from the Influenza Virus Resource refer to the NA gene segment (http://www.ncbi.nlm.nih.gov/genomes/FLU/FLU.html). (0.04 MB DOC) [file ppat.1000133.s015.doc]

**Table S5**. Influenza A viruses used in Figure S4. GenBank accession numbers and collection dates for the NA gene of 17 A/H1N1 influenza viruses sampled globally from 2006, including the A/H1N1 components of the influenza vaccines for 2006-2007 (A/New Caledonia/20/1999) and 2007-2008 (A/Solomon Islands/3/2006). GenBank accession numbers from the Influenza Virus Resource refer to the NA gene segment (<http://www.ncbi.nlm.nih.gov/genomes/FLU/FLU.html>).

| **Accession** | **Date** | **Isolate name** |
| --- | --- | --- |
| ABU99111 | 11/26/06 | A/Brisbane/297/2006(H1N1) |
| BAF63132 | 5/19/06 | A/Hanoi/BM356/2006(H1N1) |
| BAF63120 | 3/7/06 | A/Hanoi/Q137/2006(H1N1) |
| BAF63124 | 3/15/06 | A/Hanoi/Q177/2006(H1N1) |
| BAF63136 | 6/8/06 | A/Hanoi/TX09/2006(H1N1) |
| CAD57252 | 1999 | A/New Caledonia/20/1999(H1N1) |
| ABU99068 | 8/21/06 | A/Solomon Islands/3/2006(H1N1) |
| ABS71653 | 2006 | A/Thailand/CU32/2006(H1N1) |
| ABS71644 | 2006 | A/Thailand/CU41/2006(H1N1) |
| ABS71650 | 2006 | A/Thailand/CU44/2006(H1N1) |
| ABS71648 | 2006 | A/Thailand/CU51/2006(H1N1) |
| ABS71645 | 2006 | A/Thailand/CU53/2006(H1N1) |
| ABS71649 | 2006 | A/Thailand/CU57/2006(H1N1) |
| ABS71646 | 2006 | A/Thailand/CU67/2006(H1N1) |
| ABS71651 | 2006 | A/Thailand/CU68/2006(H1N1) |
| ABS71652 | 2006 | A/Thailand/CU75/2006(H1N1) |
| ABS71647 | 2006 | A/Thailand/CU88/2006(H1N1) |
