# Supplementary material for: Molecular Epidemiology of A/H3N2 and A/H1N1 Influenza Virus during a Single Epidemic Season in the United States
Source: PLoS Pathog. 2008 Aug 22;4(8):e1000133. doi: 10.1371/journal.ppat.1000133 (PMC2495036; doi:10.1371/journal.ppat.1000133)
Supplement: Table S6 — Influenza A viruses used in Figures 6– 9 and Figures S7, S8, S9, S10. GenBank accession number, isolate name, subset membership, clade membership, date of collection, week of collection, age and sex of patient from whom isolate was collected, and county in which isolate were assembled for 69 A/H3N2 influenza A viruses collected from December 6, 2006–March 14, 2007 from 16 U.S. states. Week 1 denotes the first week that isolates in this study were sampled (week of December 2, 2006). GenBank accession numbers from the Influenza Virus Resource refer to the PB2 gene segment (http://www.ncbi.nlm.nih.gov/genomes/FLU/FLU.html). (0.16 MB DOC) [file ppat.1000133.s016.doc]

**Table S6**. Influenza A viruses used in Figures 6-9 and Figures S7-S10. GenBank accession number, isolate name, subset membership, clade membership, date of collection, week of collection, age and sex of patient from whom isolate was collected, and county in which isolate were assembled for 69 A/H3N2 influenza A viruses collected from December 6, 2006 – March 14, 2007 from 16 U.S. states. Week 1 denotes the first week that isolates in this study were sampled (week of December 2, 2006). GenBank accession numbers from the Influenza Virus Resource refer to the PB2 gene segment (<http://www.ncbi.nlm.nih.gov/genomes/FLU/FLU.html>).

| **Accession** | **Isolate Name** | **Clade** | **Week of Collection** | **Date of Collection (MM/DD/YY)** | **County of Collection** | **Age of Patient (years)** | **Sex of Patient** |
| --- | --- | --- | --- | --- | --- | --- | --- |
| ABV30667 | A/Alabama/UR06-0482/2007(H3N2) | a | 13 | 3/1/07 | Columbiana | 31 | M |
| ABV30425 | A/Alabama/UR06-0545/2007(H3N2) | b | 14 | 3/7/07 | Madison | 25 | F |
| ABV30260 | A/California/UR06-0118/2007(H3N2) | s1 | 9 | 1/29/07 | Hacienda Heights | 23 | F |
| ABV29831 | A/California/UR06-0347/2007(H3N2) | a | 12 | 2/19/07 | Hacienda Heights | 48 | M |
| ABW91261 | A/California/UR06-0463/2007(H3N2) | a | 13 | 2/28/07 | Hacienda Heights | 41 | F |
| ABV45980 | A/California/UR06-0547/2007(H3N2) | a | 14 | 3/6/07 | Hacienda Heights | 8 | M |
| ABW40201 | A/California/UR06-0565/2007(H3N2) | a | 14 | 3/7/07 | Hacienda Heights | 39 | M |
| ABV45914 | A/California/UR06-0589/2007(H3N2) | b | 14 | 3/13/07 | Hacienda Heights | 48 | F |
| ABW38373 | A/Colorado/UR06-0023/2007(H3N2) | s2 | 6 | 1/8/07 | Louisville | 3 | F |
| ABW91217 | A/Colorado/UR06-0024/2007(H3N2) | a | 6 | 1/8/07 | Louisville | 2.5 | M |
| ABV29732 | A/Colorado/UR06-0206/2007(H3N2) | a | 10 | 2/6/07 | Louisville | 13 | M |
| ABW91206 | A/Colorado/UR06-022/2007(H3N2) | a | 6 | 1/8/07 | Louisville | 3 | F |
| ABW40036 | A/Colorado/UR06-0279/2007(H3N2) | a | 11 | 2/14/07 | Louisville | 9 | F |
| ABV30524 | A/Colorado/UR06-0453/2007(H3N2) | a | 13 | 2/27/07 | Louisville | 16 | F |
| ABW40091 | A/Colorado/UR06-0454/2007(H3N2) | a | 13 | 2/26/07 | Louisville | 2 | F |
| ABV30238 | A/Colorado/UR06-0531/2007(H3N2) | a | 14 | 3/6/07 | Louisville | 14 | F |
| ABW91250 | A/Colorado/UR06-0534/2007(H3N2) | a | 14 | 3/7/07 | Louisville | 6 | M |
| ABV45870 | A/Colorado/UR06-0535/2007(H3N2) | a | 14 | 3/7/07 | Louisville | 8 | F |
| ABV30084 | A/Colorado/UR06-0557/2007(H3N2) | a | 14 | 3/8/07 | Louisville | 84 | M |
| ABV45991 | A/Colorado/UR06-0558/2007(H3N2) | a | 14 | 3/7/07 | Louisville | 5 | M |
| ABV30282 | A/Florida/UR06-0150/2007(H3N2) | a | 9 | 2/2/07 | St Petersburg | 14 | F |
| ABV29721 | A/Florida/UR06-0597/2007(H3N2) | a | 14 | 3/14/07 | St Petersburg | 6 | M |
| ABV30656 | A/Illinois/UR06-0030/2007(H3N2) | a | 7 | 1/15/07 | Bloomingdale | 23 | F |
| ABV30645 | A/Illinois/UR06-0036/2007(H3N2) | a | 7 | 1/15/07 | Bloomingdale | 19 | F |
| ABV29908 | A/Illinois/UR06-0334/2007(H3N2) | a | 12 | 2/19/07 | Naperville | 59 | M |
| ABW91448 | A/Illinois/UR06-0402/2007(H3N2) | a | 12 | 2/24/07 | Bloomingdale | 29 | M |
| ABV30480 | A/Illinois/UR06-0436/2007(H3N2) | a | 13 | 2/27/07 | Naperville | 11 | M |
| ABV30216 | A/Illinois/UR06-0478/2007(H3N2) | a | 13 | 3/1/07 | Naperville | 30 | M |
| ABW40146 | A/Illinois/UR06-0528/2007(H3N2) | a | 14 | 3/6/07 | Naperville | 37 | M |
| ABW91272 | A/Illinois/UR06-0546/2007(H3N2) | a | 14 | 3/6/07 | Lake Zurich | 25 | F |
| ABV46002 | A/Illinois/UR06-0555/2007(H3N2) | a | 14 | 3/7/07 | Lake Zurich | 56 | M |
| ABV29633 | A/Illinois/UR06-0567/2007(H3N2) | a | 14 | 3/9/07 | Naperville | 35 | M |
| ABY51236 | A/Kentucky/UR06-0044/2007(H3N2) | a | 7 | 1/17/07 | Florence | 4 | F |
| ABW40333 | A/Kentucky/UR06-0158/2007(H3N2) | a | 10 | 2/5/07 | Hopkinsville | 2 | F |
| ABV30491 | A/Kentucky/UR06-0370/2007(H3N2) | b | 12 | 2/21/07 | Hopkinsville | 3 | F |
| ABV30128 | A/New York/UR06-0040/2007(H3N2) | a | 7 | 1/17/07 | Glendale | 45 | M |
| ABV30392 | A/New York/UR06-0373/2007(H3N2) | b | 12 | 2/21/07 | Bronx | 48 | M |
| ABV30227 | A/New York/UR06-0437/2007(H3N2) | a | 13 | 2/27/07 | Bronx | 24 | M |
| ABV30403 | A/New York/UR06-0510/2007(H3N2) | s3 | 14 | 3/5/07 | Bronx | 10 | F |
| ABV29919 | A/New York/UR06-0515/2007(H3N2) | s4 | 14 | 3/5/07 | Glendale | 32 | M |
| ABV30249 | A/New York/UR06-0529/2007(H3N2) | a | 14 | 3/6/07 | Glendale | 34 | M |
| ABY51038 | A/Ohio/UR06-0256/2007(H3N2) | a | 11 | 2/12/07 | Akron | 18 | F |
| ABW86573 | A/Ohio/UR06-0410/2007(H3N2) | a | 12 | 2/23/07 |  | 10 | M |
| ABW40388 | A/Ohio/UR06-0494/2007(H3N2) | a | 13 | 3/2/07 | Washington | 1 | M |
| ABW91558 | A/Oregon/UR06-0200/2007(H3N2) | a | 10 | 2/6/07 | Newberg | 7 | M |
| ABW91404 | A/Oregon/UR06-0202/2007(H3N2) | a | 10 | 2/6/07 | Newberg | 3 | M |
| ABY51137 | A/Oregon/UR06-0221/2007(H3N2) | a | 10 | 2/8/07 | Newberg | 13 | F |
| ABW40663 | A/Oregon/UR06-0272/2007(H3N2) | a | 11 | 2/13/07 | Newberg | 5 | M |
| ABW40597 | A/Oregon/UR06-0273/2007(H3N2) | a | 11 | 2/13/07 | Newberg | 6 | F |
| ABW40278 | A/Oregon/UR06-0289/2007(H3N2) | a | 11 | 2/13/07 | Newberg | 13 | M |
| ABW40641 | A/Oregon/UR06-0389/2007(H3N2) | a | 12 | 2/19/07 | Newberg | 14 | F |
| ABW40256 | A/Oregon/UR06-0450/2007(H3N2) | a | 13 | 2/26/07 | Newberg | 12 | M |
| ABW39882 | A/Texas/UR06-0356/2007(H3N2) | a | 12 | 2/20/07 | Conroe | 10 | M |
| ABW91239 | A/Texas/UR06-0358/2007(H3N2) | a | 12 | 2/20/07 | Conroe | 2 | M |
| ABV30414 | A/Texas/UR06-0418/2007(H3N2) | a | 13 | 2/26/07 | Conroe | 10 | F |
| ABV30271 | A/Texas/UR06-0480/2007(H3N2) | b | 13 | 3/2/07 | Conroe | 10 | M |
| ABV82572 | A/Texas/UR06-0566/2007(H3N2) | a | 14 | 3/9/07 | Conroe | 6 | F |
| ABY51280 | A/Texas/UR06-0603/2007(H3N2) | a | 14 | 3/14/07 | Conroe | 3 | M |
| ABW71359 | A/Vermont/UR06-0469/2007(H3N2) | a | 13 | 3/1/07 | Bennington | 5 | M |
| ABV30447 | A/Vermont/UR06-0470/2007(H3N2) | a | 13 | 2/27/07 | Bennington | 6 | F |
| ABV30073 | A/Vermont/UR06-0471/2007(H3N2) | a | 13 | 2/28/07 | Bennington | 14 | M |
| ABV30436 | A/Vermont/UR06-0483/2007(H3N2) | a | 13 | 3/2/07 | Bennington | 7 | M |
| ABV29941 | A/Vermont/UR06-0484/2007(H3N2) | a | 13 | 3/2/07 | Bennington | 7 | F |
| ABV30458 | A/Vermont/UR06-0486/2007(H3N2) | a | 13 | 3/1/07 | Bennington | 2 | F |
| ABV29820 | A/Vermont/UR06-0524/2007(H3N2) | a | 14 | 3/5/07 | Bennington | 11 | F |
| ABW36178 | A/Virginia/UR06-0021/2006(H3N2) | a | 6 | 1/8/07 | Dale City | 37 | F |
| ABW91657 | A/Virginia/UR06-0580/2007(H3N2) | a | 14 | 3/12/07 | Richmond | 6 | M |
| ABW39948 | A/Washington/UR06-0225/2007(H3N2) | a | 10 | 2/8/07 | Woodinville | 19 | F |
| ABW40179 | A/Washington/UR06-0252/2007(H3N2) | a | 11 | 2/12/07 | Woodinville | 16 | F |
