# Supplementary material for: Molecular Epidemiology of A/H3N2 and A/H1N1 Influenza Virus during a Single Epidemic Season in the United States
Source: PLoS Pathog. 2008 Aug 22;4(8):e1000133. doi: 10.1371/journal.ppat.1000133 (PMC2495036; doi:10.1371/journal.ppat.1000133)
Supplement: Table S7 — Influenza A viruses used in Figures 6– 9 and Figures S7, S8, S9, S10. GenBank accession numbers, collection dates, and age and sex of patients from whom influenza viruses were assembled for 104 A/H3N2 influenza viruses sampled globally from 2003–2006. GenBank accession numbers from the Influenza Virus Resource refer to the PB2 gene segment (http://www.ncbi.nlm.nih.gov/genomes/FLU/FLU.html). (0.17 MB DOC) [file ppat.1000133.s017.doc]

**Table S7**. Influenza A viruses used in Figures 6-9 and Figures S7-S10. GenBank accession numbers, collection dates, and age and sex of patients from whom influenza viruses were assembled for 104 A/H3N2 influenza viruses sampled globally from 2003-2006. GenBank accession numbers from the Influenza Virus Resource refer to the PB2 gene segment (<http://www.ncbi.nlm.nih.gov/genomes/FLU/FLU.html>).

| **Accession** | **Collection Date** | **Isolate Name** | **Country** | **Patient Age** | **Patient Sex** |
| --- | --- | --- | --- | --- | --- |
| ABC50430 | 3/6/05 | A/Canterbury/03/2005(H3N2) | New Zealand | 77Y | M |
| ABC67608 | 9/12/04 | A/Canterbury/109/2004(H3N2) | New Zealand | 24Y | M |
| ABD94755 | 6/29/05 | A/Canterbury/125/2005(H3N2) | New Zealand | 23Y | M |
| ABC67707 | 7/2/05 | A/Canterbury/127/2005(H3N2) | New Zealand | 28Y | F |
| ABC67718 | 7/3/05 | A/Canterbury/129/2005(H3N2) | New Zealand | 29Y | F |
| ABC67564 | 5/20/05 | A/Canterbury/16/2005(H3N2) | New Zealand | 13Y | M |
| ABC67729 | 7/8/05 | A/Canterbury/166/2005(H3N2) | New Zealand | 6M | F |
| ABC43192 | 8/13/04 | A/Canterbury/17/2004(H3N2) | New Zealand | 53Y | M |
| ABC85907 | 5/24/05 | A/Canterbury/20/2005(H3N2) | New Zealand | 17Y | M |
| ABD16570 | 7/22/05 | A/Canterbury/205/2005(H3N2) | New Zealand | 82Y | F |
| ABC85874 | 7/16/05 | A/Canterbury/220/2005(H3N2) | New Zealand | 72Y | F |
| ABD16515 | 7/27/05 | A/Canterbury/230/2005(H3N2) | New Zealand | 68Y | M |
| ABD16504 | 7/24/05 | A/Canterbury/233/2005(H3N2) | New Zealand | 12M | M |
| ABC85863 | 7/27/05 | A/Canterbury/234/2005(H3N2) | New Zealand | 42Y | M |
| ABC84530 | 7/31/05 | A/Canterbury/236/2005(H3N2) | New Zealand | 1Y | M |
| ABC85841 | 7/31/05 | A/Canterbury/237/2005(H3N2) | New Zealand | 35Y | M |
| ABC85775 | 8/1/05 | A/Canterbury/238/2005(H3N2) | New Zealand | 46Y | M |
| ABC67674 | 5/4/05 | A/Canterbury/24/2005(H3N2) | New Zealand | 54Y | F |
| ABC85830 | 8/1/05 | A/Canterbury/242/2005(H3N2) | New Zealand | 6Y | M |
| ABD16493 | 8/3/05 | A/Canterbury/248/2005(H3N2) | New Zealand | 4Y | M |
| ABD16324 | 8/16/05 | A/Canterbury/258/2005(H3N2) | New Zealand | 49Y | M |
| ABC67838 | 8/1/05 | A/Canterbury/259/2005(H3N2) | New Zealand | 28Y | F |
| ABD60822 | 5/25/05 | A/Canterbury/26/2005(H3N2) | New Zealand | 11Y | M |
| ABC67685 | 5/27/05 | A/Canterbury/29/2005(H3N2) | New Zealand | 22Y | M |
| ABC68092 | 6/1/05 | A/Canterbury/33/2005(H3N2) | New Zealand | 36Y | M |
| ABD61314 | 6/7/05 | A/Canterbury/34/2005(H3N2) | New Zealand | 16Y | F |
| ABC41702 | 7/14/03 | A/Canterbury/382/2003(H3N2) | New Zealand | 2Y | M |
| ABC42515 | 7/16/03 | A/Canterbury/420/2003(H3N2) | New Zealand | 2Y | M |
| ABC43016 | 8/17/03 | A/Canterbury/431/2003(H3N2) | New Zealand | 58Y | M |
| ABC86134 | 7/18/03 | A/Canterbury/432/2003(H3N2) | New Zealand | 70Y | M |
| ABA26776 | 9/22/04 | A/Christchurch/184/2004(H3N2) | New Zealand | 28y | M |
| AAX57652 | 10/31/03 | A/New York/11/2003(H3N2) | USA | 8y | M |
| AAX57953 | 2/4/03 | A/New York/192/2003(H3N2) | USA | 3m | M |
| AAX76742 | 2/19/03 | A/New York/193/2003(H3N2) | USA | 23y | n/a |
| AAX76752 | 2/5/03 | A/New York/194/2003(H3N2) | USA | 41y | M |
| AAY64281 | 2/19/03 | A/New York/196/2003(H3N2) | USA | 57y | M |
| AAY64301 | 3/14/03 | A/New York/197/2003(H3N2) | USA | 5y | F |
| AAY18205 | 3/8/03 | A/New York/198/2003(H3N2) | USA | 24y | F |
| AAY28627 | 3/21/03 | A/New York/199/2003(H3N2) | USA | 1y | F |
| AAY44774 | 4/10/03 | A/New York/201/2003(H3N2) | USA | 37y | M |
| AAY28324 | 4/5/03 | A/New York/202/2003(H3N2) | USA | 3m | M |
| AAY47094 | 5/7/03 | A/New York/203/2003(H3N2) | USA | 4m | F |
| AAZ83252 | 8/16/03 | A/New York/204/2003(H3N2) | USA | 39y | M |
| AAY44670 | 4/22/03 | A/New York/213/2003(H3N2) | USA | 91y | n/a |
| ABC40652 | 4/23/03 | A/New York/214/2003(H3N2) | USA | 22y | F |
| AAY28003 | 12/1/03 | A/New York/24/2003(H3N2) | USA | 9y | M |
| ABB53684 | 2/2/05 | A/New York/243/2005(H3N2) | USA | 19m | M |
| AAY64341 | 11/27/03 | A/New York/268/2003(H3N2) | USA | 32y | F |
| AAY64401 | 11/28/03 | A/New York/269/2003(H3N2) | USA | 78y | F |
| ABI22169 | 3/22/06 | A/New York/3/2006(H3N2) | USA | 49y | F |
| AAX56419 | 1/5/04 | A/New York/31/2004(H3N2) | USA | 66y | M |
| AAX56429 | 1/9/04 | A/New York/33/2004(H3N2) | USA | 1y | M |
| ABA12761 | 1/4/05 | A/New York/352/2005(H3N2) | USA | 58y | M |
| AAZ38626 | 12/21/04 | A/New York/392/2004(H3N2) | USA | 16y | M |
| ABA42301 | 1/24/05 | A/New York/395/2005(H3N2) | USA | 19y | F |
| ABG80446 | 3/22/06 | A/New York/4/2006(H3N2) | USA | 52y | M |
| AAX11544 | 12/8/03 | A/New York/42/2003(H3N2) | USA | 22y | F |
| ABB46446 | 2/2/05 | A/New York/461/2005(H3N2) | USA | 84y | M |
| ABB79741 | 2/8/05 | A/New York/462/2005(H3N2) | USA | 4m | M |
| ABB53728 | 9/20/04 | A/New York/469/2004(H3N2) | USA | 54y | F |
| ABD15503 | 12/31/03 | A/New York/474/2003(H3N2) | USA | 83y | F |
| ABD61270 | 11/19/03 | A/New York/477/2003(H3N2) | USA | 30y | M |
| ABB03122 | 1/20/03 | A/New York/485/2003(H3N2) | USA | 2y | M |
| ABG88827 | 4/5/06 | A/New York/5/2006(H3N2) | USA | 33y | M |
| AAX35830 | 2/5/04 | A/New York/52/2004(H3N2) | USA | 20y | F |
| AAY64211 | 12/16/03 | A/New York/58/2003(H3N2) | USA | 28y | F |
| AAY18135 | 12/16/03 | A/New York/59/2003(H3N2) | USA | 44y | M |
| AAY28394 | 1/6/04 | A/New York/6/2004(H3N2) | USA | 65y | F |
| ABJ53492 | 4/6/06 | A/New York/6/2006(H3N2) | USA | 15y | F |
| ABI30886 | 4/5/06 | A/New York/7/2006(H3N2) | USA | 9y | F |
| ABO32848 | 2/23/06 | A/New York/923/2006(H3N2) | USA | 13y | F |
| ABN59400 | 2/28/06 | A/New York/928/2006(H3N2) | USA | 80y | M |
| ABN51031 | 2/28/06 | A/New York/928/2006(H3N2) | USA | 80y | M |
| ABM67050 | 3/1/06 | A/New York/933/2006(H3N2) | USA | 17y | F |
| ABN51064 | 3/2/06 | A/New York/938/2006(H3N2) | USA | 11y | F |
| AAZ74561 | 11/4/04 | A/New York/98/2004(H3N2) | USA | 80y | n/a |
| ABI30619 | 7/1/05 | A/Otago/1/2005(H3N2) | New Zealand | 28 Y | M |
| ABI30842 | 8/31/05 | A/Otago/3/2005(H3N2) | New Zealand | 19 Y | F |
| ABJ16641 | 2005 | A/South Australia/23/2005(H3N2) | Australia |  | n/a |
| ABI30586 | 1/25/05 | A/Waikato/1/2005(H3N2) | New Zealand | 52 Y | M |
| ABG48224 | 6/21/03 | A/Waikato/154/2003(H3N2) | New Zealand | 4 Y | M |
| ABI30765 | 8/10/05 | A/Waikato/7/2005(H3N2) | New Zealand | 2 Y | F |
| ABI30553 | 10/19/04 | A/Waikato/72/2004(H3N2) | New Zealand | 21 Y | M |
| ABI30564 | 10/15/04 | A/Waikato/73/2004(H3N2) | New Zealand | 37 Y | F |
| ABG48268 | 1/26/04 | A/Wellington/1/2004(H3N2) | New Zealand | 56 Y | M |
| ABI21100 | 9/6/04 | A/Wellington/18/2004(H3N2) | New Zealand | 32 Y | F |
| ABI30608 | 7/11/05 | A/Wellington/2/2005(H3N2) | New Zealand | 86 Y | F |
| ABI21078 | 8/23/04 | A/Wellington/23/2004(H3N2) | New Zealand | 13 Y | F |
| ABI92422 | 7/12/05 | A/Wellington/4/2005(H3N2) | New Zealand | 55 Y | F |
| ABG80336 | 9/23/04 | A/Wellington/52/2004(H3N2) | New Zealand | 71 Y | n/a |
| ABG48279 | 8/18/04 | A/Wellington/6/2004(H3N2) | New Zealand | 23 Y | F |
| ABI30798 | 8/23/05 | A/Wellington/8/2005(H3N2) | New Zealand | 49 Y | F |
| ABI92653 | 9/9/03 | A/Western Australia/37/2003(H3N2) | Australia | 13y | M |
| ABI92708 | 9/17/03 | A/Western Australia/42/2003(H3N2) | Australia | 2y | F |
| ABI92730 | 10/22/03 | A/Western Australia/44/2003(H3N2) | Australia | 27y | M |
| ABI92796 | 9/21/04 | A/Western Australia/51/2004(H3N2) | Australia | 1y | F |
| ABI92840 | 9/14/04 | A/Western Australia/55/2004(H3N2) | Australia | 25y | F |
| ABI92862 | 10/7/04 | A/Western Australia/57/2004(H3N2) | Australia | 1y | F |
| ABI92873 | 10/1/04 | A/Western Australia/58/2004(H3N2) | Australia | 5y | M |
| ABI92928 | 10/9/04 | A/Western Australia/63/2004(H3N2) | Australia | 3y | M |
| ABJ53470 | 7/5/05 | A/Western Australia/69/2005(H3N2) | Australia | 4y | F |
| ABJ53481 | 7/21/05 | A/Western Australia/72/2005(H3N2) | Australia | 4y | F |
| ABK39983 | 8/1/05 | A/Western Australia/76/2005(H3N2) | Australia | 7m | M |
| ABK39994 | 8/3/05 | A/Western Australia/79/2005(H3N2) | Australia | 16y | M |
