# Supplementary material for: Molecular Epidemiology of A/H3N2 and A/H1N1 Influenza Virus during a Single Epidemic Season in the United States
Source: PLoS Pathog. 2008 Aug 22;4(8):e1000133. doi: 10.1371/journal.ppat.1000133 (PMC2495036; doi:10.1371/journal.ppat.1000133)
Supplement: Table S8 — A/H3N2 influenza viruses used in the 2006–2007/2007–2008 (A/Wisconsin/67/2005) and 2008–2009 (A/Brisbane/10/2007) influenza vaccines, the HA and NA gene segments of which are included in Figure 6. GenBank accession numbers from the Influenza Virus Resource refer to the PB2 gene segment (http://www.ncbi.nlm.nih.gov/genomes/FLU/FLU.html). (0.03 MB DOC) [file ppat.1000133.s018.doc]

**Table S8**. A/H3N2 influenza viruses used in the 2006-2007/2007-2008 (A/Wisconsin/67/2005) and 2008-2009 (A/Brisbane/10/2007) influenza vaccines, the HA and NA gene segments of which are included in Figure 6. GenBank accession numbers from the Influenza Virus Resource refer to the PB2 gene segment (<http://www.ncbi.nlm.nih.gov/genomes/FLU/FLU.html>).

| **Accession** | **Collection Date/Year** | **Isolate Name** |
| --- | --- | --- |
| ABW23353 | 2/6/07 | A/Brisbane/10/2007(H3N2) |
| ABW80978 | 2005 | A/Wisconsin/67/2005(H3N2) |
