# Supplementary material for: Molecular Epidemiology of A/H3N2 and A/H1N1 Influenza Virus during a Single Epidemic Season in the United States
Source: PLoS Pathog. 2008 Aug 22;4(8):e1000133. doi: 10.1371/journal.ppat.1000133 (PMC2495036; doi:10.1371/journal.ppat.1000133)
Supplement: Table S9 — Number of amino acid differences in the NA gene between clades A–H of A/H1N1 influenza virus from the 2006–2007 U.S. season. As comparison, isolates used as the H1N1 component of the influenza vaccine in 2006–2007 (A/New Caledonia/20/1999(H1N1) (NC99)) and 2007–2008 (A/Solomon Islands/3/2006(H1N1) (SI06)) are included. Amino acid differences between clades that cluster phylogenetically into set 1 (clades A–E and NC99) and clades that cluster phylogenetically into set 2 (clades F–H and SI06) are in bold. (0.05 MB DOC) [file ppat.1000133.s019.doc]

**Table S9**. Number of amino acid differences in the NA gene between clades A-H of A/H1N1 influenza virus from the 2006-2007 U.S. season. As comparison, isolates used as the H1N1 component of the influenza vaccine in 2006-2007 (A/New Caledonia/20/1999(H1N1) (NC99)) and 2007-2008 (A/Solomon Islands/3/2006(H1N1) (SI06)) are included. Amino acid differences between clades that cluster phylogenetically into set 1 (clades A-E and NC99) and clades that cluster phylogenetically into set 2 (clades F-H and SI06) are in bold.

|  | **A** | **B** | **C** | **D** | **E** | **NC99** | **SI06** | **F** | **G** | **H** |
| --- | --- | --- | --- | --- | --- | --- | --- | --- | --- | --- |
| **A** | - | 4 | 5 | 2 | 8 | 4 | **11** | **10** | **12** | **13** |
| **B** | 4 | - | 5 | 4 | 9 | 6 | **13** | **12** | **14** | **15** |
| **C** | 5 | 5 | - | 5 | 11 | 7 | **14** | **13** | **15** | **16** |
| **D** | 2 | 4 | 5 | - | 8 | 4 | **9** | **8** | **10** | **11** |
| **E** | 8 | 9 | 11 | 8 | - | 9 | **16** | **13** | **15** | **18** |
| **NC99** | 4 | 6 | 7 | 4 | 9 | - | **9** | **8** | **10** | **11** |
| **SI06** | **11** | **13** | **14** | **9** | **16** | **9** | - | 11 | 13 | 2 |
| **F** | **10** | **12** | **13** | **8** | **13** | **8** | 11 | - | 8 | 13 |
| **G** | **12** | **14** | **15** | **10** | **15** | **10** | 13 | 8 | - | 13 |
| **H** | **13** | **15** | **16** | **11** | **18** | **11** | 2 | 13 | 13 | - |
